# “Backed into a Corner”: Lived experiences of receiving and providing involuntary psychiatric treatment under British Columbia’s Mental Health Act

**DOI:** 10.1371/journal.pone.0329049

**Published:** 2026-06-10

**Authors:** Mary Elizabeth Snow, Amy Salmon, Jenyo Banjo, Marina Morrow, Colleen Varcoe

**Affiliations:** 1 Centre for Advancing Health Outcomes, Vancouver, British Columbia, Canada; 2 Faculty of Medicine, University of British Columbia, Vancouver, British Columbia, Canada; 3 School of Health Policy and Management, Faculty of Health, York University, Toronto, Ontario, Canada; 4 School of Nursing, University of British Columbia, Vancouver, British Columbia, Canada; University of North Carolina at Chapel Hill, UNITED STATES OF AMERICA

## Abstract

British Columbia’s Mental Health Act permits the involuntarily detention and treatment of individuals who meet specific criteria. Over the past 15 years, British Columbia has seen an increasing trend in the number of involuntary psychiatric admissions. This qualitative study explores the experiences of people receiving and providing involuntary psychiatric treatment within two health organizations in British Columbia, Canada. Five focus groups were conducted with 23 individuals who had previously received involuntary psychiatric treatment at a facility operated by one of the two health organizations. All sessions were facilitated by individuals with lived experience of involuntary psychiatric treatment. Additionally, semi-structured interviews were conducted with 11 clinical staff and 10 non-clinical support personnel involved in delivering involuntary psychiatric treatment or associated services. Data were analyzed using reflexive thematic analysis, guided by an equity-oriented care framework. Seven themes were generated, including the limited availability of voluntary care options, the compounding role of social determinants of health in mental health crises, the lack of conclusive evidence supporting involuntary psychiatric treatment, the negative impacts on both patients and providers, and the importance of peer support. Across themes, participants described involuntary psychiatric care as shaped by systemic constraints with limited access to upstream, voluntary and community-based alternatives. These findings highlight the need for system-level reform to reduce reliance on coercive practices and to expand access to voluntary, community-based mental health supports that address underlying social and structural factors contributing to mental health crises.

## Introduction

British Columbia’s Mental Health (MH) Act allows a person to be detained and treated without consent, including situations in which a person cannot articulate consent and in situations where a person explicitly refuses treatment — even when they are capable of making their own decisions [1]. A person can be involuntarily admitted under the authority of the MH Act to a designated facility if they meet the following criteria, known as the “involuntary admission criteria”; the person:

1. “is suffering from a mental disorder that seriously impairs the person’s ability to react appropriately to his or her environment or to associate with others”
2. “requires treatment in or through a designated facility”,
3. “requires care, supervision and control in or through a designated facility to prevent the person’s or patient’s substantial mental or physical deterioration or for the protection of the person or patient or the protection of others”, and
4. “cannot suitably be admitted as a voluntary patient.” [2]

Unlike most other jurisdictions in Canada, the BC MH Act operates on a “deemed consent” model, which assumes that once a person is detained involuntarily under the MH Act, they are deemed to have consented to any psychiatric treatment that follows [3]. The language of “in or through a designated facility” enables psychiatric treatment to be administered without a patient’s or substitute decision-maker’s consent either at a designated hospital on an in-patient basis (where the patient is detained), or in a community setting, for example, when treatment is delivered by Assertive Community Treatment teams [4,5]. Those treated involuntarily in community settings under the MH Act are referred to as being on “extended leave” from a designated facility to which they can be forcibly recalled and detained if they do not comply with the leave conditions.

The MH Act stipulates that involuntary admission status is to be used until the individual “improves to the point that they can continue as voluntary patients or resume their lives in the community.” [2]. To prevent unnecessary and indefinite detention, patients are entitled to periodic review of their detention by an independent panel [1]. However, unlike many other jurisdictions, British Columbia places the burden of requesting a review on patients who may not be fully aware of this right, or be comfortable and well equipped to make such requests [6]. Furthermore, once detained for treatment under the MH Act, there are no legal or formal procedural mechanisms available to patients to ensure oversight or review of the safety or efficacy of the psychiatric treatment administered to them, nor is there a legal mechanism for detainees to challenge psychiatric treatment administered to them against their will [7]. The only option available to patients who are concerned with their psychiatric treatment is to request a second medical opinion on the treatment’s appropriateness. There is no time limit for the opinion to be completed and the challenged psychiatric treatment may continue while the opinion is arranged [6]. Moreover, the original physician is not required to change treatment based on the second opinion.

As has been noted by BC’s Office of the Ombudsperson, the power to limit one’s liberty, bodily autonomy, privacy, and other human rights are extraordinary state powers that must be exercised rarely and with exceptional caution [8]. The province of BC currently has the highest rate of involuntary hospitalization for mental health and substance use issues in Canada [9] The rates of involuntary psychiatric admission and treatment have doubled in the last 15 years with over 20,000 people detained annually under the MH Act in BC between 2016 and 2021 [10]. During the same period, BC saw an eight-fold increase in the number of people on extended leave, i.e., receiving mandated treatment in the community [6], while a study drawing on two comprehensive systematic reviews concluded that there is no evidence of patient benefit from such practices, casting doubt over both the usefulness and ethics of these orders [11]. When population growth is considered, the use of voluntary psychiatric admissions has significantly decreased on a per capita basis [8].

Some observers have noted a correlation between increases in involuntary detainment of individuals in psychiatric crisis and limited or non-existent access to voluntary mental health treatment options, including upstream services that may prevent psychiatric crises [12–15]. Substance use disorders represented the third largest diagnosis type for people detained and involuntarily treated under the MH Act [14]. This occurs despite the fact that decades of research have failed to provide conclusive and consistent evidence supporting the effectiveness of involuntary treatments for substance use issues [16,17]. Negative outcomes have been observed pertaining to involuntary treatment of substance use disorders, including increased risk of post-release mortality [18], overdose [19], interruptions to or limited access to opioid agonist treatment [20], negative effects on therapeutic relationships, exposure to further coercive measures (including restraints), as well as disproportionate harms to specific populations [21,22]. Recent studies from BC have found that people who use drugs do not endorse the use of involuntary treatment, pointing instead to significant changes required to address shortcomings of the voluntary care system, including needs for expanded access to existing services and a wider range of harm reduction, early intervention, and treatment options [23,24].

A 2022 review by the BC Office of the Ombudsperson found that none of BC’s health authorities were fully compliant with the required documentation practices for involuntary psychiatric admissions under the MH Act [10]. To document medico-legal compliance, the MH Act mandates specific forms to be completed by health professionals at prescribed times. BC Office of the Ombudsperson noted:

For patients and their families, the lack of adequate documentation naturally raises questions about the reasons for detention. For example, could less restrictive alternatives have been used? Ultimately, the lack of documentation raises questions about whether individuals at their most vulnerable have been detained lawfully and fairly. As a result, public confidence in the system at large is jeopardized. [8]

In response, two BC health organizations undertook system-wide efforts to ensure documentation practices conform to the legal requirements for the involuntary admissions process under the MH Act, as well as efforts to improve care providers’ and patients’ understanding of the rights of people hospitalized involuntarily under the Act. At the same time, leaders and advocates within and outside these organizations recognized that demonstrating legal administrative compliance with the MH Act alone was not sufficient to understand whether or not both organizations were delivering equity-oriented care: services that are experienced by recipients as safe, destigmatizing, trauma- and violence-informed, culturally-safe, harm reduction-oriented, anti-racist, and gender-affirming [25,26]. In alignment with these efforts, a participatory, qualitative study was conducted to better understand the lived experiences of receiving and providing involuntary treatment in these organizations’ hospitals. Our aims were: (1) to document and examine, from an equity-oriented perspective, the experiences of receiving or providing involuntary psychiatric admission and treatment under the BC MH Act and (2) identify opportunities to improve care delivery during periods of psychiatric crises that align with principles of equity-oriented care. Our specific research questions were: (1) How do people who have been involuntary admitted under the BC MH Act describe their experience of receiving involuntary treatment in two BC health organizations? (2) How do clinicians and non-clinicians describe their experiences of delivering involuntary psychiatric treatment in two BC health organizations? and (3) In what ways do people with lived and living experience (PWLLE) of receiving involuntary treatment, clinicians who delivery involuntary treatment, and service providers who support PWLLE envision care delivery during psychiatry crises that better reflects equity-oriented care?

In doing so, this study contributes to public health scholarship by integrating an equity-oriented and human rights-informed analysis of involuntary psychiatric care, conceptualizing involuntary admission and treatment not only as legal and clinical interventions, but as practices shaped by structural inequities and institutional logics that structure how consent, autonomy, safety, and coercion are produced and experienced in practice, and situating Canada’s human rights obligations as an interpretive framework for evaluating these dynamics.

### Conceptual and analytic framework

Equity-oriented care refers to approaches that explicitly aim to reduce the impacts of structural inequities on health by addressing power imbalances, historical and ongoing forms of oppression, and differential access to resources within the healthcare systems. In mental health contexts, equity-oriented care integrates trauma- and violence-informed practice, cultural safety, harm reduction, and anti-racist approaches as interconnected rather than discrete components (25, 26). Guided by this framework, this study recognizes that experiences of health, illness, and healthcare are shaped by intersecting structural inequities, including colonialism, racism, poverty, gendered and sexual marginalization, and criminalization. From this perspective, involuntary psychiatric admission and treatment are understood not solely as clinical or legal interventions, but as practices that operate within broader institutional, social, and policy contexts. Experiences of consent, safety, and autonomy are therefore shaped not only by individual mental states, but also by organizational constraints, risk management logics, and the availability of voluntary and preventive services. This analytic lens allows for examination of how coercive practices may emerge even in settings where clinicians and organizations are committed to providing compassionate and ethical care. These conceptual commitments informed the study design, including the use of participatory methods, the leadership of people with lived experience in data collection, and the analytic focus on how structural conditions shape both patient and provider experiences of involuntary treatment.

## Methods

All aspects of the study were guided by an Advisory Committee comprised of people with lived or living experience (PWLLE) of receiving involuntary psychiatric treatment, clinicians who have provided involuntary psychiatric treatment, staff in community agencies supporting those receiving involuntary psychiatric treatment, clinicians and leaders working within the health organizations’ mental health and substance use services unit, and researchers with expertise in equity-oriented mental health policy and practice. The Advisory Committee was involved across all phases of the study, including protocol development, data collection planning, analytic interpretation, and reporting. This study was approved by the UBC–Providence Health Care Research Institute Research Ethics Board (REB #H22-02037).

### Reflexivity and rigour

This study was conducted by a multidisciplinary team that included academic researchers, clinicians, and people with lived experience of involuntary psychiatric admission and treatment. Consistent with the participatory orientation of the study, focus groups with people who had received involuntary psychiatric treatment were facilitated by individuals with lived experience rather than by academic researchers. This approach was intended to reduce hierarchical dynamics, support participant safety, and centre experiential knowledge as a legitimate and valued form of expertise. Academic researchers were present during focus groups but did not lead discussions.

Analytic rigour was supported through iterative coding, regular analytic meetings, and collaborative discussion of emerging interpretations. Preliminary themes were reviewed with the Advisory Committee, and areas of divergence or uncertainty were discussed with attention to how positionality, professional roles, and lived experience shaped meaning-making. These strategies were used to enhance the credibility, reflexivity, and trustworthiness of the analysis.

Qualitative data were collected via interviews and focus groups with people who have (1) received treatment under the MH Act at the two health organizations’ facilities, (2) provided treatment under the MH Act, or (3) provided legal or peer support to those who have received such treatment. Methods such as in-depth interviews and focus groups allow for rich, detailed assessment of people’s everyday experiences, providing context that is essential for crafting strategies that truly meet the needs of these populations [27,28]. Qualitative methods were prioritized for the ability to identify, from the perspective of individuals, how lived experiences are intertwined with systemic issues that contribute to health disparities, such as implicit bias in care delivery, cultural barriers to accessing services, and other factors that influence how different individuals and population groups interact with the healthcare system [29–32]. Furthermore, qualitative research fosters a deeper understanding of cultural contexts, which is essential when working with a diverse range of populations. It allows researchers to examine how factors such as personal or cultural beliefs, health-related practices, and individual and collective values influence health behaviours and perceptions of care [33–35]. These insights are critical for developing interventions and policies that address the root causes of health inequities [36,37]. This is particularly important for policymaking which aims to ensure that healthcare services are anti-racist, culturally safe, destigmatizing, trauma- and violence-informed, and responsive to the needs of all communities [26,38–41].

### Study setting

The study was conducted within two large regional health organizations in a publicly funded provincial health system. Both organizations provide integrated mental health and substance use services across urban centres and surrounding rural communities. Their service delivery spans a continuum of care that includes emergency departments, designated inpatient psychiatric facilities operating under provincial mental health legislation, and community-based mental health programs. This breadth of service provision across acute and community settings provides a contextual basis for interpreting findings and may enhance their relevance to other similarly structured health systems.

### Data collection

#### Focus groups.

Advisory Committee members with lived experience of involuntary psychiatric admission and treatment strongly recommended that qualitative data collection with people who have received such treatment would best be conducted in a focus group setting. Participants were recruited between February 1 and April 30, 2023, through social media and community agencies that work with PWLLE of involuntary psychiatric admission and treatment. Interested individuals were asked to contact a member of the research team for eligibility screening. Eligible participants had to be 19 years of age or older and have a history of involuntary psychiatric admission and treatment at any of the facilities operated by the two health organizations within the past five years.

To accommodate participants’ preferences, two focus groups were held in person and three were conducted virtually. Because these organizations operate healthcare facilities in urban and rural settings, one of the virtual focus groups was specifically for participants from rural communities. Attendance at the focus groups ranged from 2 to 5 participants and a total of 23 people participated. All participants provided informed written consent to participate in the study; however, only 6 participants consented to provide information about their gender or age. At the advice of research team members with lived experience, collection of demographic information was intentionally limited and optional due to concerns regarding confidentiality, safety, and the potential for re-identification in a small and highly stigmatized population. Consistent with participatory and trauma- and violence-informed research principles, participants were not required to disclose demographic details to participate in the study, and many chose not to do so. While this limits the ability to describe the sample in demographic terms, it reflects participant preferences and ethical priorities in this research context. Of the 6 participants who provided demographic information, 4 (66.7%) identified as female. The mean age was 49.5 years (SD = 15.6; range: 25–63 years). One participant (16.7%) self-identified as Indigenous.

Focus groups were designed to minimize re-traumatization, prioritize participant agency, and acknowledge structural violence embedded in mental health systems. Thus, in-person focus groups were held in community locations that were not healthcare facilities. Private space outside the focus group room was available to allow in-person participants to step away from the group for some time if needed. Breakout rooms were made available during virtual focus groups for the same purpose. In both the in-person and virtual breakout rooms, a research team member was available to provide emotional and social support if requested. All focus groups were facilitated by one or two PWLLE with support from two research team members with qualitative research experience. Focus groups lasted 1.5 to 2 hours each, were audio recorded and transcribed verbatim. The focus group guide is available in S1 Data‌‌ Collection Tools.‌‌

#### Interviews.

Advisory Committee members from health professions recommended that semi-structured interviews with those who provide treatment or support to individuals who have received admission and treatment under the MH Act be conducted individually. Participants were recruited between February 1 and April 30, 2023, through purposive sampling of individuals who provide services to people admitted for involuntary psychiatric treatment under the MH Act. Participants were purposively sampled based on their professional roles and locations of work to ensure diverse representation. Interview participants provided informed written consent.

Two researchers with qualitative interview experience (AS and MES) conducted interviews with 21 individuals. Table 1 shows participants’ roles. Interviews lasted approximately 1 hour each, were conducted virtually, audio recorded, and transcribed verbatim. The interview guide is available in S1 Data Collection Tools.

**Table 1:** Categories of participants who provide clinical care and non-clinical support.

Category	Role	Number of participants
Clinician	Nurse	3
	Physician	5
	Social Worker	3
Service Provider^a^	Lawyer/Legal Advocate	4
	Peer Support Worker	4
	Program Leader/Manager	2

*Note.* ^a^ The term “service provider” is used to refer to professionals providing non-clinical support for individuals admitted and treated involuntarily under the BC MH Act.

### Ethical considerations

Research focused on involuntary psychiatric treatment presents heightened ethical considerations due to the potential for re-traumatization and the context under study. Informed consent was approached as an ongoing, relational process rather than a single event. Participants were reminded of their right to pause or withdraw at any time without consequence.

To support participant well-being, focus groups were designed using trauma- and violence-informed research principles. Sessions were held in community locations rather than healthcare settings where possible. Breakout rooms were available during both in-person and virtual focus groups, allowing participants to step away or speak privately with a research team member if they experienced distress or required a break. These supports were offered proactively and without requirement to disclose reasons.

Peer facilitators were supported by research team members with qualitative research experience, and post-session check-ins were available as needed. All identifying details were removed from transcripts, and findings are presented in aggregate to protect confidentiality.

### Data analysis

Data were analyzed using a thematic analytic approach using NVivo (version 14) guided by an equity-oriented care framework [26] to organize the data and track the analysis. The framework’s components of safety, stigma, trauma- and violence-informed, cultural safety, harm reduction-orientation, anti-racism, and gender affirmation served as sensitizing concepts that informed our interpretation of themes. Analysis of qualitative data from individual interviews and focus groups followed the steps articulated by Braun and Clarke [42,43] for conducting reflexive thematic analysis:

1. Familiarization. Multiple research team members, including those who conducted interviews and those who had not participated in the data collection process, thoroughly read and re-read the data, and then began identifying initial concepts or patterns generated in the narrative accounts.
2. Coding. Two members independently reviewed transcripts and reflexive notes generated in the familiarization stage to generate concise, descriptive, inductive codes for segments relevant to the study aims. To centre the voice of PWLLE, the focus group transcripts were coded first, followed by coding the interview transcripts. Codes captured discrete elements of participants’ experiences, including life-saving treatment, peer support, desire for autonomy, resource constraints, and perceived threat. Iterative meetings were held to discuss interpretive differences, refine codes and develop a shared analytic understanding. Final codes reflected repeated elements across the dataset while preserving the nuances of diverse perspectives. For example, PWLLE and clinicians described involuntary treatment as stressful or uncomfortable, but their interpretations diverged: clinicians emphasized safety, legality, and resource constraints, whereas PWLLE highlighted loss of autonomy and emotional distress.
3. Generating Themes. Codes with conceptual similarity were grouped into subthemes, which were then clustered into overarching themes representing broader patterns across the dataset. Themes represent inductive interpretations of participant accounts, while the equity-oriented care framework was used at the interpretive level to situate findings within the broader and systems-level implications. Theme development was guided by the equity-oriented care framework [26], which served as an interpretive lens at this stage of the analysis. Examining the codes through this lens revealed the presence or absence of safety, stigma, trauma- and violence-informed care, cultural safety, harm reduction orientation, anti-racism, and gender affirmation, which allowed themes to reflect the significance of the themes in relation to equity-oriented care during times of psychiatric crisis. Analytic decisions prioritized both prevalence and conceptual significance, ensuring that aligned and divergent experiences across participants groups were retained.
4. Reviewing Themes. Generated themes were presented to the Advisory Committee along with illustrative examples from the coding process. Committee members reviewed the themes to confirm that they accurately reflected the material present in the data set. Areas of divergence or uncertainty were discussed collaboratively, considering the influence of researcher positionality on interpretation.

To demonstrate transparency and traceability, Table 2 presents illustrative excerpts alongside their corresponding codes, subthemes, and themes, including examples from PWLLE, clinicians, and non-clinical service providers. This table highlights the pathway from raw data to higher-order interpretation, showing how aligned and divergent perspectives were preserved while capturing the complexity of involuntary psychiatric treatment experiences.

**Table 2:** Illustrative excerpts with corresponding codes, subthemes, and themes.

Excerpt	Participant	Codes	Subtheme	Theme
“If I was really not connected to reality or wanting to end my life, I would be, later not in the moment, thankful that I did have involuntary treatment because it potentially could have saved my life. I just wish there was a different process for going through it all like we’re sharing now, with options and stuff.”	PWLLE	Bittersweet experience; Life-saving treatment; Desire for autonomy	Ambivalent treatment experience; Restricted autonomy	Theme 2: Stress, trauma, and distrust; Theme 4: Reduced patient autonomy (all-or-nothing consent)
“People go back to their lives that were the same as when they came in…hospital policies could be changed to include outside entities…continuity of care until they’re well enough to be on their own.”	Clinician	Lack of continuity; Need for external support; Policy gaps; Revolving door care	Systemic barriers to care	Theme 1: Lack of voluntary, community-based care; Theme 5: Limited treatment options
“In emergency, people…get thrown in seclusion because nurses don’t have time…nurses see it as preserving safety…most clinicians are doing their best.”	Clinician	Provider discomfort; Resource constraints; Safety concerns	Provider constraints; Ethical tensions	Theme 2: Stress, trauma, and distrust; Theme 4: Reduced patient autonomy (all-or-nothing consent); Theme 5: Limited treatment options; Theme 7: Provider distress
“The thing that has helped me most was having a friend come with me to Emergency…she was at least there…be a moral support, peer support.”	PWLLE	Peer support; Emotional safety; Stress alleviation; Trust	Supportive care experiences	Theme 2: Stress, trauma, and distrust; Theme 3: Peer support improves experience
“I’m escorted by security guards on a wheelchair…if a peer support worker was walking alongside me…that would help me get comfortable and know what to expect.”	PWLLE	Perceived threat; Peer support; Emotional reassurance; Stress alleviation; Orientation support; Desire for autonomy	Supportive care experiences	Theme 2: Stress, trauma, and distrust; Theme 3: Peer support improves experience; Theme 4: Reduced patient autonomy (all-or-nothing consent)
“I think it’s environment too. When you think about patients…on a stretcher being assessed and then, involuntarily certified, you would just never want that for someone you care about.”	Clinician	Provider discomfort; Resource constraints	Provider constraints; Ethical tensions	Theme 2: Stress, trauma, and distrust; Theme 5: Limited treatment options; Theme 7: Provider distress
“In my experience, there is no test for mental illness. There’s no scan, no blood work…you come into many facilities, through Emergency and you get put in their quiet room, or their seclusion room…if your needs fit in with what works for the staff, OK. But if your needs don’t fit in with their process, then their process wins every time.”	Service provider	Subjective assessment; Power imbalance; Escalation due to rigid rules; Trauma triggers	Structural/procedural constraints; Restricted autonomy	Theme 2: Stress, trauma, and distrust; Theme 4: Reduced patient autonomy; Theme 5: Limited treatment options

## Findings

Seven themes were generated, listed in Table 3.

**Table 3:** Thematic findings from interviews and focus groups with patients and providers.

Theme
1. Lack of voluntary, community-based mental healthcare within the context of the social determinants of health leads to involuntary psychiatric admissions and complicates discharges
2. Involuntary psychiatric admission and treatment is a stressful experience for both people receiving and providing treatment and can lead to harm, trauma, and distrust in the healthcare system
3. Peer support improves experience of care
4. An “all or nothing” consent approach reduces patient autonomy and restricts rights
5. Available treatments during involuntary admission are limited to a narrow range of options
6. Stigma and discrimination occur
7. The lack of conclusive evidence for involuntary treatment creates distress for care providers

### Theme 1: Lack of voluntary, community-based mental healthcare within the context of the social determinants of health leads to involuntary psychiatric admissions and complicates discharges

Among the most salient themes found in both the interview and focus group data was the view that voluntary treatment, both in community to prevent people from reaching a state of mental health crisis and in hospital for those requiring acute care, is extremely limited and inaccessible to many. PWLLE and clinicians emphasized that community-based mental health supports are insufficient to meet demand, with programs often unavailable and unaffordable, and long wait lists and strict entry requirements making it hard to qualify for services.

We shouldn’t have to prove, actively prove in front of them, that the situation is bad enough to actually get treatment. – PWLLE

We don’t have enough acute psychiatric resources in the system, and we also don’t have enough at the other end. We don’t have enough places for people that have serious mental health dual diagnosis issues who don’t need to be in hospital but don’t have a place to go. – Clinician

The perspective of PWLLE and clinicians was that available mental health supports provided in community are insufficient to meet needs of people in this geographic region. This contributes to circumstances in which people reach a state of mental health crisis and perpetuates reliance on acute care. Both groups of participants assessed that, from a pragmatic perspective, the only meaningful way for many people to receive timely access to mental healthcare is to be admitted involuntarily to hospital. Interview participants reported that most people who receive psychiatric treatment in hospital are admitted involuntarily (with the exception of people receiving treatment for eating disorders, who participants observed tend to be predominantly treated voluntarily).

People are uncomfortable with voluntary patients in an acute care setting … it almost feels like a luxury to be admitted voluntarily in an acute care setting. – Clinician

Even where preventative services are available, the stigma and ableism associated with “mental illness,” and the consequences of seeking help for such readily stigmatized conditions, were mentioned as barriers to accessing services. This in turn was reported to have negative implications for other determinants of health, such as employment.

My employer has no idea that I have a mental illness. I cannot divulge. I would get degraded in my work, if not even let go – kind of pushed under the carpet – and one day I’m gone… So there needs a lot more done to also support people that are like me, living and hiding and have nowhere to run. - PWLLE

The role of social determinants of health — including housing, transportation, income, social support, food, spirituality, and culture — on mental wellness was also emphasized by PWLLE, clinicians, and other service providers. PWLLE reported their conditions are exacerbated by unmet basic needs, leading to mental health crises and repeat involuntary admissions. Additionally, for those on extended leave, not having access to key social determinants of health can contribute to not being able to meet the conditions of extended leave (e.g., attending appointments), ultimately leading to recall to hospital.

People go back to their lives that were the same as when they came in. – Clinician

Somebody’s required to make an appointment and to show up on a day, or they’re getting recalled and going to the hospital. If that person is sleeping on the side of [the road], doesn’t have a clock, doesn’t know what day it is, doesn’t have the support to get there, and they missed their appointment with their mental health team. [Then] it’s negative documentation about their not following through, they’re not doing ABC and D. They’re getting recalled back to the hospital and they get put in same cycle again. – Clinician

Some clinicians noted that, in some cases, involuntary psychiatric admission and treatment was the only avenue for patients to access (or continue to access) resources to meet their basic needs. Clinicians frequently remarked that they felt “backed into a corner” to certify patients under the MH Act due to a lack of system-level support for voluntary care and community-based service coordination to address social determinants of health.

I feel like that we get a little bit more backed into a corner to do involuntary treatment. Because I think we know that without it, sometimes this person might lose all these resources that they have, and it will be very hard to get them back. If our system was different, and people could get housing, and they could try again somewhere else, I think we would maybe feel less, or at least I would feel less, kind of stuck in the need for involuntary treatment in this context. – Clinician

Furthermore, clinicians described the challenges of working within an acute care setting focused on getting patients out of hospital quickly, with limited capacity to promote care continuity. The acute care system was seen by these participants as not being conducive to delivering quality care and supporting people post-discharge with access to supports promoting mental well-being. They also described the distress it caused them when they must discharge patients knowing there are few to no low-barrier, accessible resources to which to refer them.

Usually, they give you a little slip of paper that says (sometimes not always), sometimes a slip of paper that says you can do this, that and the other thing. But you’re kind of left [on your own]. Sometimes it will say that you have a follow-up appointment but that only happened to me once. Otherwise, it’s just goodbye, good luck. And it’s terrifying going from the secure environment of a hospital back into the real world… especially if you live alone. It’s very scary. – PWLLE

They just get discharged when they are good enough, you know, but they’re not. They’re just good enough to go home and not be taking up a $1,600 a day hospital bed. – Clinician

Clinicians described how a lack of resources in community even for those on extended leave can be a barrier to discharging a patient from hospital and can lead to patients having repeated episodes of involuntary psychiatric admission and treatment.

Some of the more intensive mental health resources require that people have tried and failed different things. So, sometimes we know we’re setting someone out on extended leave, probably to a mental health service that’s not gonna meet their needs, and that it’s probably not gonna be successful and it’s going to lead to them being recalled, brought in by the police. – Clinician

These accounts indicate that involuntary psychiatric admission frequently operates as a downstream response to gaps in voluntary and preventive services, with structural constraints shaping both crisis trajectories and post-crisis care pathways.

### Theme 2: Involuntary psychiatric admission and treatment is a stressful experience for both people receiving and providing treatment and can lead to harm, trauma, and distrust in the healthcare system

PWLLE described a spectrum of experiences with involuntary psychiatric treatment. Some indicated that, due to their mental state while in crisis, they would not have sought or accepted care voluntarily, and, looking back, were grateful to have received treatment.

For me, at the onset of my illness, only outside intervention and getting me into hospital seems to work. I don’t think I would have actually survived, if I would have continued roaming on the street and not eating. – PWLLE

However, it was unclear whether the benefit was ascribed to the involuntary circumstances under which treatment was delivered, or if a similar outcome would have been achieved if some or all aspects of their care were voluntary.

I am thankful that I did have involuntary treatment… I just wish there was a different process for going through it all like we’re sharing now, with options and stuff. – PWLLE

Some described a range of negative experiences during involuntary psychiatric admission.

Participants repeatedly stated the use of coercive measures, including the understanding they were being “stripped of their rights” or being treated “like a criminal” when in hospital under the MH Act, led to fear and overwhelm, feeling stigmatized and traumatized. Some participants described feeling disempowered and being made to feel “like a child”.

But if somebody like me gets stripped of all their rights and put into seclusion rooms — and close the door and injected and have no idea what’s going on — that’s a pretty brutal assault to one’s human dignity, to one’s human rights, to one’s perception of who we are in the community, how we are appreciated and included in a greater community. – PWLLE

And I feel we are treated and under the law, we are [treated like a] criminal because our rights are taken away. We’re not criminals. We’re in a medical facility but we’re treated as a criminal that has done something wrong. – PWLLE

You begin to feel like you’re being treated like a child when you’re actually an adult with a mental illness, which is very different than being a child. – PWLLE

PWLLE discussed how these negative experiences of involuntary psychiatric admission and treatment impacted them, including creating lasting trauma and lowered self-esteem. Some felt fearful of reliving the traumatic experience, which, in turn, discouraged them from seeking healthcare or fully disclosing their concerns to clinicians in the future. Health professionals corroborated this, noting they have witnessed ways that trauma associated with involuntary psychiatric admission and treatment can deter people from seeking care, both for mental and physical health concerns.

Now for future, if I need to go back, I know I can’t be honest. I have to filter what I say or I’m going to be in a very scary situation that is going to, that is traumatic. And yeah, it’s taught me not to be honest when I seek mental healthcare. – PWLLE

It also impacts access to healthcare because a lot of my clients will not bring themselves to the hospital or to a clinic if they’re having a health issue, because they’re afraid of being re-certified again. – Service Provider

Another consequence of involuntary psychiatric admission and treatment is that it can become a system barrier for patients to receive other forms of care and support, including accessing onsite programming at hospitals while admitted. For example, if a patient is in seclusion, they are unable to access other programs or services during that period.

The involvement of police in apprehending people under the MH Act was highlighted by PWLLE as a significant source of trauma, and by clinicians as compromising the therapeutic relationship. Experiencing, witnessing, or being aware of circumstances in which people experiencing mental health crises were “tasered”, “attacked”, handcuffed, or “dragged” by police while being detained and transported for involuntary treatment complicated and constrained participants’ willingness to engage with health services. For those participants, this created additional barriers to access. For some clinicians, police involvement created unnecessary complexities for healthcare delivery, creating additional strain on their ability to assess and care for patients in emergency settings.

Since I was having an episode, so I decided to run instead of being safe and being like, to stand here. So, [the officer] attacked me, put me in cuffs. – PWLLE

Separation between healthcare and police is super important… I don’t think police are trained or suited to make those [mental health] assessments. And in my experience, they’re the ones escalating the situation. – Clinician

A lot of the times, the police will bring somebody, and they’ll say “look, we’re just told to bring them in. I have no idea why, like literally none.” I have to deal with it right? I’m responsible so those stress me out quite a bit. There’s no information whatsoever. The patient looks perfectly normal. There’s no supporting documentation why certain decisions got made. – Clinician

The majority of clinicians expressed a range of negative feelings related to involuntarily admitting and treating patients, including feeling burned out, traumatized, and inadequate due to working in a system that limits their ability to meet their patients’ needs. For instance, emergency department clinicians described the environment as unconducive to providing care to people experiencing a mental health crisis. The chaotic environment, lack of privacy, and lack of time to interact with patients limited their ability to deliver quality care.

I think it is environment too. When you think about patients in Emergency on a stretcher being assessed and then involuntarily certified, you would just never want that for someone you care about. – Clinician

I have to interview people in the parking lot, because every single care space is full, like literally. Everything is full. So, we have to go on the freezing parking lot. – Clinician

Clinicians felt hindered by not knowing a patient’s history and having limited time to observe a patient or establish a relationship. This was stated to increase the likelihood of activating “triggers” for PWLLE that may exacerbate mental health crises and determining a course of action that might not be of maximum benefit to the person they were treating.

One of the things for example is the observation period. If the patient [within] two hours is completely rational, that’s an alteration period as well, where I’m going to be a lot less likely to do something. But if given only the five-second observation period to make decision, I’ll probably err on the side of certifying somebody. – Clinician

Some participants described adversarial feelings between clinicians and patients, between different clinicians, and between lawyers representing patients and clinicians, which can introduce additional risks and harms to all involved. For example, one participant commented:

There are some [clinicians] that are great and have no problem with their patients, clients, whatever, applying for Review Panels. Others take that quite personally – that their judgment is being questioned – that makes it difficult. – Service Provider

Risks and fears discussed by clinicians included the conflicting fears that if they do not involuntarily detain someone, they will be responsible if “something bad happens”, and fears that if they do involuntarily detain someone, they could be held liable or sued. Clinicians also mentioned the risk of harm to staff by patients when attempting to deliver treatment against their patient’s will. Many participants talked about involuntary psychiatric admission and treatment being traumatic for patients, introducing additional emotional harms.

These findings show that involuntary care is experienced as both a clinical and relational intervention, with significant emotional and institutional consequences that can erode trust in healthcare systems for both patients and providers.

### Theme 3: Peer support improves experience of care

Many PWLLE felt having a peer to help them navigate the system while being admitted and treated would have been of tremendous benefit in allaying their fears and improving their experience.

Another thing that would really, really help is peer support. Having [someone] there that could reassure me that, “OK this is what’s going to happen, you’re going to meet with the psychiatrist” or whatever the process is. And then if I’m admitted into the ward, or in this case certified, to explain what that means. – PWLLE

Clinicians agreed that peer support roles were valuable; however, they noted peer support workers are often treated as though they are not part of the care team with negative consequences for people in these roles.

[Peers] had a really significant role in building relationships and helping people to access care. But the peers had a hell of a hard time, and we went through so many of them because they were stepping into an environment where there was fierce hierarchy. They didn’t even have letters after their names; they weren’t going to be taken seriously, and your self-esteem can only handle so much. – Clinician

Moreover, both PWLLE and interview participants felt including patient advisors with lived and living experience in planning services would provide valuable expertise for developing more holistic programs and services.

Another source of support identified by PWLLE was engagement and inclusion of family and friends. However, PWLLE had varied views regarding if and to what extent they would have liked their families to be informed about or involved in their involuntary psychiatric admission and treatment. Some participants felt family members could use more education on how to support their loved ones who are experiencing mental health crises. Others identified that family members, including chosen family and others providing support from within their communities, could serve as a valuable source of information to clinicians as they deliver care to people in crisis.

Peer support appears to play a stabilizing relational role in involuntary care by providing continuity, reassurance, and system navigation, although its impact is constrained when insufficiently integrated into clinical teams.

### Theme 4: An “all or nothing” consent approach reduces patient autonomy and restricts rights

PWLLE described a lack of control over all aspects of their treatment, and some reported they were not informed of what was happening — including not being informed of their rights under the MH Act. An “all or nothing” phenomenon seems to occur with the BC MH Act deemed consent approach, where once a person is involuntarily admitted under the MH Act, there is a default that all treatment and related interventions are delivered involuntarily. This approach leaves no room for patients to state their preferences or have those preferences considered in treatment planning.

I think the hardest part about it was I felt like a persona non grata. I felt like nobody discussed anything with me. – PWLLE

Nobody told me what was happening. Nobody told me that I had any rights, they just gave me two shots of Loxapine and I went to sleep. – PWLLE

[I was told] “you’re here for shock treatment” and then they explained what that all meant. They did very good in that respect in explaining what shock treatment was, and even I got to watch a video about it. So that part was good. But no, there was no “Oh! by the way, do you want to get this?” – PWLLE

PWLLE described the importance of connection and compassion. Whenever they experienced empathy and compassion from clinicians or felt listened to and involved as much as possible in their care, this had a positive impact on their well-being and their engagement in care.

Any time I’ve had a doctor or care provider that has included me in the decision-making or at least made it feel that way, I’ve found that the outcomes have been better and I’m more willing to take the medication. – PWLLE

They also noted that clinicians are very busy and burnt out, and it is challenging for staff to have enough time to meaningfully engage with their patients.

Some PWLLE emphasized information should be shared with patients in an easy-to-understand way and when they are more receptive.

But the rights [under the MH Act] are so much, there is a lot of jargon, so it doesn’t really make sense to you, especially if you’re not in the right mindset to listen. – PWLLE

Participants also noted that when a person treated under the MH Act is informed of their rights, there are many process barriers to acting on this knowledge. For example, participants noted a potential for conflict when clinicians providing care are the ones to inform patients of their rights under the MH Act, including the right to a second medical opinion and a review panel. Moreover, they were concerned about the potential for a conflict of interest to arise when seeking second medical opinions, as the second physician consulted often works on the same care team as the first physician. In addition, there was concern there is no requirement for the first physician to change the treatment based on the second opinion. Concern was also expressed that when a person treated under the MH Act challenges their treatment at a review panel hearing, it is often a unit clerk who selects which health record documentation to provide to the lawyer representing the patient. In these circumstances, participants expressed concern that unit clerks may not have sufficient medical and legal knowledge to determine which parts of a patient chart are relevant. Another concern was that clinicians sometimes had to play roles during review panel hearings that impair their relationship with their patients.

[It] creates a very weird dynamic, because they are acting kind of as counsel, they are also the expert witness; and, of course, they’re the client’s treating doctor. So, it can really impair the treatment relationship for it to proceed in that way. – Service Provider

These experiences suggest that the deemed consent model is often enacted in practice as a withdrawal of patient participation, with limited opportunities for meaningful involvement in care decisions during involuntary admission.

### Theme 5: Available treatments are limited to a narrow range of options

When reflecting on options available for “treatment” to those being treated under the MH Act, medication, physical restraints, and seclusion were often mentioned. References to non-pharmaceutical treatment options, such as psychotherapy, tended to focus on how it is lacking. Some clinicians raised concerns about over-dependence on the use of physical and chemical restraints, as well as seclusion. A pertinent recommendation from one interview participant was to institute frequent re-assessments of patients in seclusion and hold debriefing sessions after every seclusion event to identify ways things could have been done differently. Clinicians and PWLLE alike expressed desire for meaningful and timely access to a much broader array of options, including counsellors and psychologists; recreation, music, and art therapy; and access to the outdoors.

In emergency, people get thrown in seclusion because the nurses don’t have time, or they feel they don’t have time, or they’re worried that they’re going to abscond, or whatever, and they kind of lock them in there in an unethical way. But I don’t think that the nurses see it that way. They see it as they’re preserving their safety, whereas the patient probably wouldn’t feel that way. – Clinician

There are so many things to do in response to someone yelling and only one of them is putting them in seclusion. – Service Provider

Treatment options during involuntary admission are constrained by institutional capacity and workflow pressures, resulting in reliance on a narrow set of interventions across acute care settings. This theme was most commonly discussed by clinicians and service providers, reflecting how this issue was discussed across participant roles in the dataset.

### Theme 6: Stigma and discrimination occur

Those who provide care and support to people who experience involuntary psychiatric admission and treatment noted some instances of discrimination, such as clinicians’ perceptions of risk and behaviour being influenced by racism (e.g., men of colour viewed as more dangerous) and sexism (e.g., women being judged on their appearance and sexual behaviour).

I can tell you that I have unquestionably seen anti-Indigenous racism, sexism, ableism, operating in the attitudes of the people enforcing detention involuntary treatment which has changed the way the experience has played out for my clients. Yes, that’s what I think I could sort of confidently say. – Service Provider

I have been treated like somebody that doesn’t have any life or nothing together, and [later] I had gone through university, had a professional degree and actually have been working as a professional, suddenly I was treated differently. And the doctor, constantly commenting on my university degree and on my education. – PWLLE

There were also mentions of people being certified during pregnancy over concern for the fetus and then decertified once the baby was delivered. In addition, there were discussions of challenges for birthing parents who are involuntarily treated, as there is currently no option for them to have their newborns with them during an involuntary admission in these organizations’ facilities.

There’re people who have been using [substances] and don’t necessarily have mental health challenges that I would assume would warrant certification in the hospital, but because they’re pregnant, that becomes the designated reason as to why they’re certified. – Service Provider

Examples of anti-Indigenous racism and discrimination against transgender people were also provided. For example, an interview participant shared that they had seen a patient chart that described an Indigenous patient who was speaking their language as speaking “gibberish.” As another example, interviewees described situations where a patient’s identification with a gender different than the one assigned to them at birth was viewed as a symptom of mental illness. In these examples, the lack of culturally safe or gender-affirming care is not only discriminatory, but clinicians were using patients’ behaviour in response to this discrimination as “evidence” of a mental health issue.

The person recently is identifying as male and it says right in the report, the doctor’s handwritten notes, “This person likes to go by – the name that this person has chosen is Dave and the pronouns are he/him.” And it’s mentioned in handwritten notes a couple times that, “The client – patient – was upset and didn’t feel heard or understood.” And the physician referred to [the persons as] “her” consistently in handwritten notes, in the case note, and in the entire presentation. That’s not uncommon, that’s just one example I think of medicalizing and, “This person is delusional,” that kind of stuff. – Service Provider

Indigenous people serving in the role of liaisons for Indigenous patients in the health organizations were noted as providing culturally safe care and interview participants expressed a desire for people serving in this type of role. Also mentioned was the added layer of complexity in which Indigenous patients are held without their consent in a place where they have no access to cultural healing and wellness practices, language-speakers, or other tangible aspects of culturally safe care.

There was concern that equity-oriented care and related principles — such as cultural safety, patient-centred care, harm reduction, gender-affirming, and trauma-informed practice — are buzzwords that are not translated into practice. Conscious, conspicuous, and transparent efforts are needed to ensure these principles are applied to practice within the context of involuntary psychiatric admission and treatment. This includes capacity for ongoing monitoring and evaluation focused on understanding how these efforts are experienced by care recipients and care providers, and what the tangible results are over time.

I don’t think you can actually be trauma-informed when the policies and the system itself is just perpetuating that trauma. So, yeah, it feels a bit like an oxymoron. – Service Provider

These accounts highlight how stigma and discrimination are embedded within clinical and institutional practices, shaping both decision-making and interpretations of patient behaviour.

### Theme 7: The lack of conclusive evidence for involuntary treatment creates distress for care providers

When considering the above issues together, and in light of their lived experience providing care, participants who have dedicated their careers to supporting people during mental health crises raised many questions and concerns about the lack of evidence for involuntary psychiatric admission and treatment. In so doing, they reflected openly on whether the presumed “benefits” of using the MH Act to intervene with someone experiencing a mental health crisis are substantiated by available evidence, and whether such approaches justify the harms they repeatedly observed occurring for their patients and clients when they are subjected to involuntary and coercive “care”. These participants expressed concern that little is known about the long-term impacts of removing a patient’s rights on their health and well-being, and about the financial impacts of continuing with “a carceral approach” to mental health service delivery at times when health systems are increasingly under pressure economically and related to adequate staffing levels.

For some, the experience of being asked to mandate care of unknown quality with unknown results was inconsistent with their goals to ensure all patients receive high-quality, evidence-based care. These participants advocated strongly for a transformation in how mental health crisis response is delivered, centring preventive, community-based, and voluntary supports that acknowledge and respond to social determinants of health and inequities in service access and treatment outcomes.

Some of my concerns about this field, which seems unique to this field, is that the intention to help shuts down any evaluation about what the impact is… I’m not aware of any good evidence on evaluating what the long-term benefits or harms etc. are for involuntary intervention, so that’s something that feels problematic and unique to this area. – Service Provider

When you mandate care that you know is good quality, then it’s one thing. If you mandate care that it’s unclear what the positive effect will be other than in the acute period, that’s very problematic or that brings out the more problematic aspect of mandated care which is terminating of freedom that a person has. – Clinician

I know how expensive a carceral approach is, I know how much the police costs and I know how much having someone locked up in a psych ward cost. So yeah, I’d like to see a world where voluntary services are not only available (which doesn’t exist, so that’s a huge step), and then where the voluntary services are something that’s incentivized and that people feel like they want to go. – Clinician

Clinicians’ reflections reveal how uncertainty regarding the effectiveness and long-term impacts of involuntary treatment contributes to ethical tension and professional distress. These concerns highlight the challenges of delivering mandated care in contexts where evidence is limited and where providers are simultaneously accountable for patient safety, legal compliance, and quality of care. This theme was most commonly discussed by clinicians and service providers, reflecting how this issue was discussed across participant roles in the dataset.

In summary, our findings demonstrate that, overall, participants in this study did not experience involuntary psychiatric admission and treatment under the MH Act in ways that are consistent with the principles of equity-oriented care (i.e., care experienced as safe, destigmatizing, trauma- and violence-informed, culturally-safe, harm reduction-oriented, anti-racist, and gender-affirming) [25,26]. PWLLE, clinicians, and other professionals who support PWLEE highlighted the stressful and often traumatic nature of their experiences with involuntary psychiatric admissions and treatment. PWLLE discussed feeling afraid, overwhelmed, stigmatized, and disempowered. Concerns were also raised about treatment received being limited to primarily medication, restraints, and seclusion, and challenges to accessing their rights to second medical opinions and review panels. Moreover, participants expressed the view that a lack of voluntary, prevention-oriented, community-based mental health services and unmet basic needs exacerbate mental health issues, leading to a reliance on involuntary psychiatric admission and treatment. This lack of community-based services was also reported to be challenging with efforts to provide adequate discharge planning. Practices that were experienced as helpful included peer support, and instances where PWLLE felt listened to and involved as much as possible in their own care.

## Discussion

These findings demonstrate that both those who receive and deliver mental health services under BC’s Mental Health Act can experience multiple, systemic, and interrelated barriers to implementing equity-oriented care. We identified many factors mediate these experiences, which require urgent attention to move towards a more equity-focused approach in alignment with commitments to safeguarding human rights. This study offers contributions to the growing body of empirical literature examining patient and provider experiences of delivering mental health services via involuntary care policies.

While many of the substantive findings align with existing literature on involuntary psychiatric admission and treatment, this study contributes context-specific evidence from a “deemed consent” legal framework within two large regional health organizations operating across urban and rural settings. The inclusion of multiple perspectives (people with lived experience, clinicians, and legal/peer support roles) also enables a more integrated account of how involuntary care is experienced across different roles within the system. In addition, the participatory design of the study supported richer interpretation of participants’ accounts by incorporating input from people with lived experience throughout data collection and analytic interpretation, strengthening the contextual grounding and credibility of the findings.

*The experience of involuntary treatment:* As in this study, research from other jurisdictions has found that in the context of involuntary psychiatric admission and treatment, people are subjected to chaotic and frightening care environments [44,45], coerced medication administration as a “taken-for-granted” practice [45], and being treated like children [15,45,46] or prisoners [44]. Qualitative accounts elsewhere further affirm the perspectives of participants in this study: if they had been listened to and had their needs respected during the time of crisis, treatment could have occurred without coercive measures [45].

Involuntary psychiatric admission and treatment has been described elsewhere in the literature as traumatizing [45,47], frightening [44], confusing [47], and stigmatizing [44,46], negatively impacting trust of healthcare providers [48], and leading people to avoid care in the future [45,49]. Similarly, studies have found that the experience of providing involuntary psychiatric admission and treatment has a negative emotional and psychological impact on healthcare professionals [48].

*Factors that support positive experiences in involuntary mental health treatment settings:* Some studies have shown that people who have experienced involuntary psychiatric admission and treatment feel they have benefited from their treatment, and others have documented perspectives that participants felt they would not have accepted treatment otherwise [15,46,50]. Similar to the participants in this study, reports elsewhere have noted study participants views that their primary concern was not with the need for psychiatric retreatment, but that they would have preferred to receive treatment through voluntary means with opportunities for fully informed decision-making [15,44,50].

The importance of clinicians listening to, understanding, and respecting patients [15,46] to build trust to improve the quality of care during psychiatric treatment [45] is a common theme in the literature on involuntary psychiatric treatment. Participants in this study noted that making choices is important to their recovery and being involved in decision-making about their care could increase as their recovery progresses [44]. Others have also documented similar recommendations, including the critical importance of listening to patients, building trusting relationships, as well as developing individualized, flexible care and discharge plans in cooperation with patients [45]. Alongside participants in this study, authors elsewhere have also described the importance of patient’s having access to advocates [15,44] and peer support workers [44] in hospital and community settings. Such supports can enhance opportunities to improve communication between patients and providers, assist patients to articulate their needs and preferences, and open doors for sharing experiential knowledge that supports system navigation and empowerment when engaging in treatment. This is particularly true when such efforts are integrated with commitments to anti-racist, decolonizing, culturally safe care (63).

*The need for voluntary treatment modalities and integrated health and social care:* As in this study, previous research describes medication [15,44] and restraints [15] as being the main interventions used during involuntary psychiatric admission and treatment, with little use of other interventions that could be beneficial, such as psychotherapy and meaningful recreational, educational, or occupational activities [44,46]. Moreover, participants emphasized the desire for more early voluntary services to address mental health concerns before a crisis begins [49,51], stressing the importance of the role of social determinants of health in precipitating or exacerbating to mental health crisis [47,51]. It has been well demonstrated that a lack of preventative mental healthcare and compromised social determinants of health such as inadequate affordable housing, experience of broader structural inequities, and discrimination contribute to involuntary psychiatric admission and treatment [47]. Attention to social determinants of health when considering mental health service needs, and opportunities for service and system responses that integrate health and social care are of tremendous importance in this context.

Some interview participants in this study expressed concern that there is limited evidence about whether involuntary treatment is effective, and this concern is also echoed in the literature. In a scoping review on ethical issues in clinical decision-making about involuntary psychiatric treatment, Laureano et al. [49] note that “the lack of data on the clinical benefit of involuntary hospitalization has been identified as a serious limitation” and from a systematic review on involuntary psychiatric hospitalization and long-term compliance, Cossu et al. [52] concluded that “although evidences carried out so far are weak, the data do not show a trend of improvements and do not seem to exclude the possibility of worse compliance after compulsory hospitalisation” [49]. Similarly, prior systematic reviews found no evidence of patient benefits from community treatment orders [11].

*Involuntary mental health treatment experiences in populations experiencing inequality and structural violence:* Participants in our study spoke of witnessing treatment of Indigenous patients that were aligned with the findings of the In Plain Sight report [53] which detailed that Indigenous people are subject to racist, discriminatory, and culturally unsafe practices. Similarly, clinician participants in our study spoke of treatment of transgender patients that aligned with previous studies showing that gender diverse and transgender people experience anti-transgender bias, non-affirmation, and further trauma when seeking mental healthcare [54,55]. Previous research has shown that members of groups experiencing oppression and discrimination, such as Indigenous and racialized groups and young people, are impacted in numerous ways by coercive practices in mental healthcare [56]. As highlighted by Morrow and Weisser (2012) [57] “although involuntary detention of First Nations, Métis, Inuit and urban Indigenous children and youth under the MH Act may be intended for their safety and protection, it can be seen and experienced as another link in a long chain of oppression imposed by the state on Indigenous peoples” (p. 4). The “In Plain Sight” report similarly identified emergency departments and psychiatric services as settings in which Indigenous peoples in BC are subject to racist, discriminatory, and culturally-unsafe practices, including being detained involuntarily without clear rationale or for reasons informed by anti- Indigenous racism and stereotyping [58].

There is also ample literature attesting to the ways in which structural violence in the form of patriarchal, heteronormative, and transphobic practices have shaped, constrained, and undermined access to timely, appropriate, and supportive mental healthcare for women, gender-diverse people, and 2SLGBTQ+ communities, while normalizing violence and coercion in psychiatric treatment settings [59–62]. Recent research has confirmed that gender-diverse and transgender people experience greater inequities in many dimensions of mental wellbeing, which are in turn often compounded by anti-transgender bias, non-affirmation, and further trauma when seeking mental healthcare [54,55]. An Ontario study reported that expectations of negative or demeaning treatment in healthcare cause transgender people to avoid care to the extent that only 29 percent of transgender respondents seek healthcare for any reason [63]. Of those who accessed emergency care, 52 percent reported experiencing “negative” (transphobic) treatment ranging from insulting or demeaning language to outright refusal of care [63].

*Equity-oriented mental health care and Canada’s obligations under the UN Convention on the Rights of Persons with Disabilities:* This is not the first study in which patients receiving involuntary psychiatric treatment report not being provided information about the reasons for their detention [15,46] or their rights [44,47]. Globally, movements for health justice and health equity are emphasizing the critical importance of aligning mental health service provision to international commitments to promote well-being and safeguard human rights [64,65]. Canada is a signatory to the 2006 United Nations Convention on the Rights of Persons with Disabilities (UN CRPD), which requires signatory countries to undertake substantive reforms to protect and promote human rights in mental health services, and to ensure a continuum of community-based supports are available to prevent conditions which result in institutionalization of all kinds. Article 19 of the UN CRPD requires signatory States to recognize “the equal right of all persons with disabilities to live in the community, with choices equal to others” and to “take effective and appropriate measures to facilitate full enjoyment by persons with disabilities of this right” [66]. This includes the right to “have access to a range of in-home, residential and other community support services, including personal assistance necessary to support living and inclusion in the community, and to prevent isolation or segregation from the community” [66]. As noted by the UN Special Rapporteur on the Rights of Persons with Disabilities in her End of Mission Statement following her 2019 visit to Canada, involuntary psychiatric admission and treatment in BC are in contradiction to articles 14 and 25 of the CRPD, including the “absence of procedural guarantees, the lack of alternative treatment options” and insufficient monitoring of designated facilities [67]. She further noted that there is a “lack of comprehensive responses to guarantee the access of persons with disabilities to the support they need to live independently in their communities” [67]. The participants in our study expressed perspectives that align with this. The UN Special Rapporteur also called on governments to provide support, including adequate housing, and for the meaningful participation of people with disabilities in decision-making.

Recent interventions in BC policy debates have emphasized the importance of ensuring BC’s mental health laws and services align with Canada’s international commitments. For example, advocates have urged efforts to bring the BC Mental Health Act “up to date with evidence-based best practices and human rights principles” [68], emphasizing needs for a holistic approach to mental wellness which prioritizes intersectional equity, supports self-determination, and meaningfully involves PWLLE in all aspects of decision- making that impacts them. The findings of this study underscore the importance of aligning mental healthcare delivery with these guiding principles.

### Strengths and limitations

This study was guided by an Advisory Committee comprised of individuals with lived and living experience of receiving or providing involuntary psychiatric admission and treatment, service providers in community agencies supporting those receiving care under the MH Act, clinicians and leaders working within the health organizations’ mental health and substance use services, and researchers with expertise in equity-oriented mental health policy and practice. We used a trauma-informed approach to the data collection and focus groups for PWLLE were led by PWLLE of involuntary psychiatric admission and treatment.

Following the Committee’s advice, we recruited participants for the focus groups for PWLLE through both social media and through community agencies that work with PWLLE. Interested participants were later screened to exclude those who did not receive involuntary treatment in the specific health organizations in which the study was situated. We did not recruit directly through the health organizations or their services directly. As part of our recruitment process, and to ensure transparency, we disclosed that this study was commissioned by the health organizations, and that results would be communicated directly to it. Consequently, we may have not reached those experiencing the greatest structural vulnerabilities when interacting with health systems, or those whose experiences of trauma during their interactions with health systems would have discouraged them from participating in a study involving those health organizations.

This is a study with a small sample size using qualitative methods, and as is typical with qualitative research of this type, results may not be generalizable to other contexts or representative of all patient or provider experiences or perspectives. While the interview guide and recruitment materials were neutral with respect to the type of experiences sought, the overwhelming majority of participants in both the treatment recipient and the care provider groups (clinician and service providers) spoke of negative, stressful, or concerning experiences. No participants spoke of experiences that were primarily positive. As a result, this study is not able to comment on policies, practices, or contexts which participants associated with positive care experiences. There was a high rate (66%) of people who registered for the in-person focus groups who did not attend the focus group; however, this did not occur in virtual focus groups, in which all who registered attended. In addition, 17 PWLLE participants did not disclose their age, gender, or other demographic information, preventing the opportunity to conduct intersectional analysis of these experiences.

## Conclusions

As is demonstrated by the above findings and supported by an ample body of evidence in other settings and jurisdictions, compliance with the administrative requirements of the MH Act alone as encouraged by BC’s Ombudsperson’s report [8] does not in itself guarantee quality of care, alignment with commitments to equity-oriented care, or the protection of human rights. The delivery of evidence-based mental healthcare in all forms requires health system and service planners to acknowledge mounting evidence within the international context, alongside Canada’s international commitments, that insist upon urgent movement away from coercive care practices. People with lived and living experience of receiving and providing involuntary admission and treatment who participated in this study identified a serious and concerning lack of system-level supports and pathways for prioritizing and enabling low-barrier access to voluntary, community-based mental health supports and for addressing the social determinants of health that often precipitate mental health crises.

## Supporting information

S1 Data Collection Tools. (PDF)
