# Supplementary material for: “Backed into a Corner”: Lived experiences of receiving and providing involuntary psychiatric treatment under British Columbia’s Mental Health Act
Source: PLoS One. 2026 Jun 10;21(6):e0329049. doi: 10.1371/journal.pone.0329049 (PMC13252785; doi:10.1371/journal.pone.0329049)
Supplement: S1 Data — (PDF) [file pone.0329049.s001.pdf]

Focus Group Guide – PWLLE

**Topic**	**Details**
**Introduction (Large Group)**	**Thank you for agreeing to participate in this focus group.**<br>• [Facilitators introduce themselves)<br>• Explain role of support person to provide information and assistance to anyone in the session who would like support.<br>• Remind participants that:<br>○ their participation is completely voluntary, they do not have to answer any questions that they do not want to, and they can leave or take a break at any time,<br>○ everything said in the focus group will be kept anonymous, and that their identities will not be disclosed in any reports.<br>**Purpose**<br>• The purpose of this focus group is to learn from people who have experienced involuntary treatment under the Mental Health Act at VCH and PHC.<br>• We want to learn from your perspective about your experience and about how these organizations can provide the best possible care during times of crisis.<br>• We will be conducting several focus groups like this and will be sharing what we learn with leaders at VCH and PHC so that they can make changes.<br>• We won't be sharing anyone's names/other identifiable information with them.<br>• We also hope to share this information with other places that can use this information to improve things, such as the Ministry of Health, the BC Office of the Ombudsperson, and the BC Office of Human Rights Commissioner.<br>• We will be asking questions about your experience with involuntary treatment, about how you would like people to be treated when you access medical services when experiencing a mental health crisis, and any recommendations you have to improve these services. The focus group will last around an hour with eight questions which will give us about 7 min per question.

	• This focus group will be audio-recorded. The recording will be transcribed to ensure we capture what everyone says in the focus group; no one other than the evaluators and a transcriptionist will listen to the recording.<br>• Ask if anyone has any questions before the focus group starts]<br>**Distribute honoraria**<br>**Split into two groups**
**Icebreaker**	• **Briefly** introduce yourself with you name or pseudonym and tell us what your favourite food is?
**Comfort Agreement**	• Confidentiality<br>• Make space, take space. One person speaks at a time.<br>• Respect diverse view and listen to learn.<br>• Be mindful of time.<br>• Take breaks to support your wellness<br>• **Anything that anyone would like to add?**
**Opening Question**	• What is your understanding of the Mental Health Act?
**Priority Question**	• Tell us about your experience of treatment without consent under the Mental Health Act?<br>o Topics to be discussed (if participants do not bring these up, you can probe about them):<br>▪ where you received involuntary treatment<br>▪ situations that lead to being treated involuntarily<br>▪ interactions with healthcare providers when being treated involuntarily<br>▪ interactions with other professionals during times of crisis (e.g., police, social workers, etc.)<br>▪ being informed (or not) about your legal rights<br>▪ loved ones being informed (or not) that you have been detained in the hospital<br>▪ being able to challenge your detention (or not)<br>▪ support or follow-up needed when discharged from the hospital<br>▪ extended leave<br>▪ effect of involuntary treatment on you? on communities?<br>o Do you recall having conversations with the doctor about your treatment (e.g., the purpose of medications, risks)? Were you made aware when there were changes to your treatment plan?
**Priority Question**	• In your experience, what has helped you most when experiencing a mental health crisis?<br>o Did you or someone supporting you contact services? If so, which ones?<br>• Why was this helpful? What were you able to do as a result of getting this help?
**Priority Question**	• What do you think access to health services should look like for people experiencing a mental health crisis? Why?

<table>
<tr><td></td><td></td></tr>
<tr><td>Priority Question</td><td>Do you think there is a role for involuntary treatment when providing services to people in crisis? Why or why not?</td></tr>
<tr><td>Priority Question</td><td>What recommendations do you have around treatment that is provided during a mental health crisis?
[Based on what things were discussed in the previous sections, can ask questions to probe about the issues the participants brought up in that discussion]</td></tr>
<tr><td>#1</td><td>Is there anything you wish had happened when you were experiencing a mental health crisis that did not happen?</td></tr>
<tr><td>#2</td><td>Are there things about the Mental Health Act that should be changed? If so, what?</td></tr>
<tr><td>#3</td><td>What is important when it comes to delivering respectful mental health care?</td></tr>
<tr><td>Closing</td><td>Ask if they have any questions? (4 min – max)

Capturing Thoughts
Is there anything else you would like to talk about that we haven’t talked about yet? If so, please take a few minutes to write down any note or email us after the session.
Next Steps
We have an Advisory Group for our project that includes people with lived experience of involuntary treatment, as well as providers, advocates, and researchers.
We will be taking all of our findings to this group to help us determine how best to share the findings.
Let us know if you would like to be contacted once the group decides what this will be.
Thank participants 😊</td></tr>
</table>

Interview Guide - Professionals

Introduction:

Thank you for agreeing to participate in this interview

[Facilitators introduce self. Remind participants that their participation is completely voluntary and they can leave or take a break at any time, their identities will not be disclosed in any reports.

We will be asking questions about your experience with providing care and/or support for patients being treated involuntarily under the Mental Health Act. The interview will last around 1hrs.

[Inform participants that the interview will be audio-recorded. The recording will be transcribed to ensure we capture what the participant says; no one other than the evaluators and a transcriptionist will listen to the recording. Ask if they have any questions before the interview starts]

Questions:

- What is your role in providing care to/supporting patients during times of psychiatric crisis
  - Probes:
    - How long have you worked in your role?
    - Where do you provide care/support?

Barriers, facilitators, challenges, and successes that they have observed or experienced in their efforts to support people experiencing psychiatric crisis

- Tell me about your experience with providing mental health support (or support to patients) during times of psychiatric crisis.
  - Probes:
    - What leads to patients being treated involuntarily?
    - What are interactions with patients like when they are being involuntarily treated? How does it differ from those being voluntarily treated?
    - How often do you provide care to/support patients for receiving involuntary treatment under the Mental Health Act?
    - What support or follow-up is provided to patients who are treated involuntarily, if any, after discharge?
    - What barriers do you experience to providing effective mental health treatment (or support to patients) during times of psychiatric crisis?
    - What helps you to provide effective mental health treatment (or support to patients) during times of psychiatric crisis?
    - What challenges and/or success have you seen in your efforts to support people experiencing psychiatric crisis?
    - What are your views about involuntary treatment?

Practices, policies, and related factors that mediate access to effective mental health treatment during times of psychiatric crisis

- What is the role of policy in shaping how people can access care, or provide care, in times of crisis or during an emergency? In your view, what are the most important ways that policies shape access to mental health care?
- Are there any policy or practice differences you are aware of between the context you work in, and approaches taken in other places?
- Do these policies and/or practices have any unique consequences for specific individuals/ patient groups? If so, what are they?
- Have you been involved in changing any policy or practice related to mental health care? What have those changes been? Why are these changes important?

Understanding of the relationship between involuntary mental health treatment practices and [organization] goals for equity-oriented care

- The [organization] has a commitment to providing equity-oriented care. What does "equity-oriented care" mean to you? Do you see examples of equity-oriented care in your day-to-day experience? If so, what are they? If not, why do you think this is the case?
- What do you think access to medical services should look like for people experiencing a mental health crisis? Why? Do you think involuntary treatment, as it is currently practiced, is compatible with goals for equity-oriented care? If so, how? If not, why not?
- Is there anything you wish had happened when you are working with a patient experiencing a psychiatric crisis that does not currently happen? Any things you wish would not happen that do?
- What recommendations do you have for treatment that is provided during a mental health crisis?
    - Probes:
        - Do any existing services, policies, or practices need to change? Why?
        - Are there services, practices, or resources aren't currently available that you wish were available?

- Is there anything else you would like to talk about that we haven't talked about yet?

Closing:

[Thank participants for their time. Ask if they have any questions]
