# Supplementary material for: “Backed into a Corner”: Lived experiences of receiving and providing involuntary psychiatric treatment under British Columbia’s Mental Health Act
Source: PLoS One. 2026 Jun 10;21(6):e0329049. doi: 10.1371/journal.pone.0329049 (PMC13252785; doi:10.1371/journal.pone.0329049)
Supplement: Attachment — Submitted filename: Reviewer Report.pdf [file pone.0329049.s003.pdf]

Reviewer Report

Manuscript title: "Backed into a Corner: Lived experiences of receiving and providing involuntary psychiatric treatment under British Columbia's Mental Health Act"

Manuscript number: PONE-D-25-37120

Dear Dr. Banjo and colleagues,

Thank you for the opportunity to review the revised version of your manuscript. I appreciate the thoughtful and substantive way in which you engaged with the previous feedback. The revisions have clearly strengthened the paper.

The addition of a clear conceptual framework significantly improves the manuscript. Equity-oriented care is now presented as an analytic lens rather than a set of descriptors, and the integration of structural determinants, colonialism, and power dynamics provides stronger theoretical grounding. The study's contribution to debates on coercion and structural inequities is much clearer.

Methodologically, the new Reflexivity and Rigour and Ethical Considerations sections enhance transparency and credibility. Your discussion of participatory processes, power dynamics, and trauma-informed safeguards meaningfully addresses prior concerns. The justification for limited demographic data is also well explained.

The analysis is now more interpretive, particularly in linking participants' experiences to systemic constraints and institutional logics. While some themes remain slightly more descriptive than others, the overall analytic depth has improved considerably. A final tightening of thematic synthesis paragraphs could further enhance coherence.

The Discussion is more cohesive and layered, with clearer policy and structural implications. A brief explicit statement of the manuscript's theoretical contribution, and, if feasible, a short link to Canada's human rights obligations, could further sharpen the paper's impact.

Overall, this is a substantially improved manuscript that makes an important and timely contribution to scholarship on involuntary psychiatric treatment and equity-oriented mental health reform.

Thank you again for the opportunity to review this meaningful work.

Lina Cuadrado MD, MSc
