# Supplementary material for: “Backed into a Corner”: Lived experiences of receiving and providing involuntary psychiatric treatment under British Columbia’s Mental Health Act
Source: PLoS One. 2026 Jun 10;21(6):e0329049. doi: 10.1371/journal.pone.0329049 (PMC13252785; doi:10.1371/journal.pone.0329049)
Supplement: Attachment — Submitted filename: Reviewer Report.docx [file pone.0329049.s002.docx]

**Reviewer Report**

**Manuscript title:** "Backed into a Corner: Lived experiences of receiving and providing involuntary psychiatric treatment under British Columbia’s Mental Health Act"

**Manuscript number:** PONE-D-25-37120

**Decision recommendation:** Minor revision

**Summary**

This manuscript addresses a highly relevant and ethically charged issue: the lived experiences of individuals receiving and providing involuntary psychiatric treatment under British Columbia’s Mental Health Act (MH Act). The topic is both timely and socially critical, particularly given the increasing use of coercive psychiatric interventions in Canada and the international attention to human rights–based mental health reform.

The authors adopt a qualitative, participatory design, guided by an advisory committee that includes people with lived experience (PWLLE), clinicians, and advocates. This participatory orientation is a major strength, as it aligns with the study’s equity-oriented framework. The data are rich, the participants’ voices are powerful, and the findings highlight systemic tensions between clinical practice, human rights, and structural inequities.

However, despite its potential, the manuscript in its current form remains descriptive rather than analytical, and methodologically underdeveloped in several key respects. Greater conceptual clarity, methodological transparency, and theoretical depth are needed to elevate it to publication standards.

**Major Comments**

**1. Conceptual framing and theoretical depth**

While the manuscript refers to “equity-oriented care,” “trauma- and violence-informed,” “culturally safe,” and “anti-racist” approaches, these concepts are not clearly defined nor theoretically integrated. The paper risks presenting them as buzzwords rather than analytic lenses.

**Recommendation:** Clarify how these frameworks informed the design, data collection, and interpretation. Explain whether the analysis was guided by a specific theoretical approach (e.g., intersectionality, critical mental health studies, decolonial theory). A brief conceptual framework section early in the paper would strengthen the argument substantially.

**2. Methodological rigor and reflexivity**

Although the participatory design is commendable, the description of how reflexivity, positionality, and analytic rigor were maintained is insufficient.

- How were power dynamics between researchers, PWLLE, and institutional staff navigated?
- Was there member checking or peer debriefing?
- How were divergent interpretations handled?

**Recommendation:** Include a Reflexivity and Rigor subsection explaining how the research team’s composition and lived experiences shaped the analysis. Consider adding a short table summarizing strategies for ensuring credibility and trustworthiness (e.g., triangulation, iterative coding, Advisory Committee validation).

**3. Data analysis and interpretation**

Thematic analysis is appropriate, but the resulting themes read as extended summaries rather than interpretive insights. The manuscript currently catalogs participants’ experiences but stops short of offering a conceptual synthesis or theoretical contribution.

Example: The “all-or-nothing consent” theme could be deepened using frameworks of biopower, governance, or medical paternalism.

Similarly, the “lack of voluntary options” theme could be connected to neoliberal health reforms or deinstitutionalization failures.

**Recommendation:** Move beyond description. Clarify what these findings mean for understanding coercion and equity, not just that they exist. Consider developing a conceptual figure linking structural determinants, coercive practices, and experiential outcomes.

**4. Ethical and epistemic considerations**

The study addresses deeply traumatic experiences. While it notes ethical approval, it lacks reflection on how risks of re-traumatization, coercion, or confidentiality were mitigated.

**Recommendation:** Add an Ethical Considerations section discussing the emotional risks for participants, how informed consent was practiced meaningfully in a coercive context, and how peer facilitators were supported.

**5. Discussion and originality**

The discussion largely repeats results and lists literature that confirms them, without building a distinctive argument.

**Recommendation:** Reorganize the discussion into three clear layers:

- Empirical: What this study adds to the evidence base in BC.
- Theoretical: How it extends debates on coercion, rights, and structural determinants.
- Policy/Practice: Concrete implications for reform (e.g., alternatives to involuntary detention, peer inclusion models, community-based prevention).

The paper could also more explicitly connect its findings to Canada’s obligations under the UN Convention on the Rights of Persons with Disabilities, analyzing not only that contradictions exist but how and why they persist.

**Minor Comments**

Participant demographics are extremely limited. Even minimal data (gender, ethnicity, years since admission) would improve transparency.

Several sections could be condensed by removing duplicated findings in Results and Discussion.

**Overall Assessment**

This is an important, well-intentioned, and ethically engaged piece of qualitative research that amplifies the voices of individuals too often silenced in mental health policy. However, it requires some revisions to achieve analytic depth, conceptual clarity, and structural coherence.

**Recommendation:**

Minor Revision: promising but requires conceptual and analytic strengthening before publication.
