# Supplementary material for: “Backed into a Corner”: Lived experiences of receiving and providing involuntary psychiatric treatment under British Columbia’s Mental Health Act
Source: PLoS One. 2026 Jun 10;21(6):e0329049. doi: 10.1371/journal.pone.0329049 (PMC13252785; doi:10.1371/journal.pone.0329049)
Supplement: Attachment — Submitted filename: 2025-12-20 Response to Reviewers.docx [file pone.0329049.s004.docx]

# Response to Reviewer

**Manuscript title:** "Backed into a Corner: Lived experiences of receiving and providing involuntary psychiatric treatment under British Columbia’s Mental Health Act"

**Manuscript number:** PONE-D-25-37120

We thank the reviewer for their thoughtful, careful, and constructive feedback. We appreciate the reviewer’s recognition of the importance of the topic, the participatory orientation of the study, and the strength of the empirical material. In response, we have revised the manuscript to improve conceptual clarity, methodological transparency, analytical depth, and ethical reflexivity, while maintaining a measured tone and a lived-experience centred approach. Below, we respond to each major comment in turn.

## Reviewer Comment 1: Conceptual framing and theoretical depth

*The manuscript refers to equity-oriented, trauma- and violence-informed, culturally safe and anti-racist approaches, but these concepts are not clearly defined or theoretically integrated.*

**Response:** We agree that clearer articulation of the conceptual framing strengthens the manuscript. We have added a Conceptual and Analytic Framework subsection in the Introduction to explicitly define equity-oriented care and clarify how trauma- and violence-informed, culturally safe, harm-reduction, and anti-racist approaches function as interconnected analytic lenses rather than as descriptive terms alone (pp. 7, lines 137-155). We situate involuntary psychiatric treatment within broader structural, institutional, and policy contexts, with attention to power relations and resource constraints. This framing clarifies how these concepts informed the study design and analytic interpretation without introducing additional theoretical frameworks.

## Reviewer Comment 2: Methodological rigour and reflexivity

*Greater transparency is needed regarding reflexivity, power dynamics, and analytic rigour.*

**Response:** We appreciate this feedback and have strengthened the Methods section accordingly. We added a Reflexivity and Rigour subsection that details the multidisciplinary composition of the research team and the role of people with lived experience in study governance, data collection, and interpretation (pp. 8-9, lines 166-180). We clarify that focus groups with people who had received involuntary psychiatric treatment were facilitated by individuals with lived experience rather than by academic researchers, with the intention of reducing hierarchical dynamics and centering experiential knowledge. We also describe strategies used to support analytic rigour, including iterative coding, collaborative analytic meetings, and Advisory Committee review of preliminary themes.

## Reviewer Comment 3: Data analysis and interpretation

*The findings are rich but remain largely descriptive; the manuscript would benefit from greater interpretive depth.*

**Response:** To strengthen analytic depth while preserving the Results as empirically grounded, we added brief analytic bridge sentences at the end of each theme (pp. 19, lines 397-402; pp. 25, lines 524-528; pp. 27, lines 563-568; pp. 30, lines 628-632; pp. 31, lines 655-658, pp. 34, lines 718-722; pp. 36, lines 763-767). These additions explicitly connect participants’ accounts to broader institutional and structural constraints, such as resource limitations, consent governance, and organizational constraints, without altering participant quotations or the structure of the Results. These revisions clarify what the findings contribute to understanding coercion and equity in practice, while maintaining the participant-centred presentation of the data.

## Reviewer Comment 4: Ethical and epistemic considerations

*The manuscript would benefit from greater reflection on ethical risks and mitigation.*

**Response:** We added an Ethical Considerations subsection to the Methods (pp. 12-13, lines 257-270). This section describes how informed consent was approached as an ongoing, relational process; the use of peer facilitators; the availability of breakout rooms during focus groups to support participant well-being; and additional trauma- and violence-informed strategies used to mitigate and protect confidentiality. These additions clarify how ethical risks were addressed in a study focussed on experiences of coercion and trauma.

## Reviewer Comment 5: Discussion and originality

*The Discussion largely repeats the Results and could be strengthened by clearer articulation or empirical, theoretical, and policy contributions.*

**Response:**

We appreciate this feedback and have revised the Discussion section to strengthen analytic synthesis and originality by integrating the study’s findings with prior empirical research, examining factors that support or constrain equity-oriented care, and situating the results within broader structural, policy, and human rights contexts (pp. 37-43, lines 786-923).

## Minor Comment: Participant demographics

*Participant demographics are extremely limited.*

**Response:** We have clarified in the Methods that the collection of demographic information was intentionally limited and optional due to concerns related to confidentiality, safety, and potential re-identification in a small and highly stigmatized population (pp. 10-11, lines 218-225). This approach aligns with participatory and trauma- and violence-informed research priorities. We acknowledge this as a limitation of the study.

## Additional Editor Comments

**Response:** We prepared the revised manuscript in accordance with PLOS ONE’s formatting and submission guidelines. We provided a *Data Availability Statement* through the submission system, including a non-author institutional contact and details on long-term data storage, in line with PLOS ONE’s Data Policy.

We clarified in the Results that coercion was experienced not only through formal detention but also through less visible practices such as exclusion from treatment planning, disregard for expressed wishes, and limited opportunities for shared decision-making (pp. 30, 626-628).

With respect to the recommended CRPD-related references, we reviewed these works and considered their relevance. At this stage, we chose not to expand the CRPD framing beyond its current scope, as doing so would require broader consultation with the author team regarding the framing of international legal guidance. The manuscript already engages substantively with Canada’s obligations under the CRPD in relation to involuntary psychiatric treatment, and we would be open to revisiting this discussion in a subsequent revision if requested.

We thank the reviewer again for their insightful feedback, which has helped strengthen the manuscript’s conceptual clarity, methodological transparency, and analytic contribution. We believe these revisions enhance the paper while remaining faithful to its participatory, equity-oriented foundation and to the voices of participants.
