# Supplementary material for: “Backed into a Corner”: Lived experiences of receiving and providing involuntary psychiatric treatment under British Columbia’s Mental Health Act
Source: PLoS One. 2026 Jun 10;21(6):e0329049. doi: 10.1371/journal.pone.0329049 (PMC13252785; doi:10.1371/journal.pone.0329049)
Supplement: Attachment — Submitted filename: 2026-04-22 Response to Reviewers.docx [file pone.0329049.s005.docx]

Response to Reviewers

| **Reviewer Comments** | **Our Revisions** |
| --- | --- |
| A final tightening  of thematic synthesis paragraphs could further enhance coherence | Thank you for your suggestion. We reviewed the thematic synthesis and made minor edits to improve clarity but did not undertake further tightening as the existing synthesis already balances coherence with preservation of analytic depth across complex qualitative themes. |
| A brief explicit statement of the manuscript’s theoretical contribution, and, if feasible, a short link to Canada’s human rights obligations, could further sharpen the paper’s impact | Thank you for this insightful suggestion. In response, we have revised the Introduction to include a clear and explicit statement of the study’s contribution to public health scholarship (p. 7, lines 146-151). This now specifies that the study extends an equity-oriented and human rights-informed analytic lens for understanding involuntary psychiatric care, conceptualizing involuntary admission and treatment as practices shaped by structural inequities and institutional logics that structure experiences of consent, autonomy, and safety in practice. We have also added a more explicit linkage to Canada’s human rights obligations within this statement, situating these obligations as an interpretive framework for analyzing involuntary psychiatric care. This revision helps foreground the relevance of human rights considerations earlier in the manuscript, while maintain more detailed discussion later in the paper (pp. 46-47 lines 944-977). |
| Clarity of the objectives and conceptual framework The study’s objectives are broad and combine description, critical analysis, and policy-oriented recommendations. It would be helpful to clarify: i) the specific research question(s), ii) how the “equity-oriented care” framework was operationalized within the analysis, and iii) the distinction between empirical findings and normative positions.  For example, the manuscript states: “Our aims were: (1) to document and examine, from an equity-oriented perspective…”, yet it does not describe how this framework guided coding, interpretation, or theme development | i) We’ve added the specific research questions to more clearly delineate the study’s empirical focus (p. 7 lines 137-144).  ii) We have further elaborated on our analysis process to clarify how the equity-oriented care framework was operationalized. Specifically, we now state that the framework informed interpretation through its core components which were used as sensitizing concepts during analysis. We also clarify that coding was primarily inductive, with the framework guiding interpretation and theme development rather than imposing a deductive coding structure (pp. 14-16 lines 301-340).  iii) We have also clarified the distinction between empirical findings and normative interpretation by specifying that themes were generated inductively from participant accounts, while the equity-oriented care framework was used to support higher-level interpretation and discussion of structural implications (pp. 15-16 lines 324-335). |
| Sampling, sample composition, and transferability. The absence of demographic data for most participants with lived experience limits the possibility of conducting an intersectional analysis, despite the manuscript’s emphasis on this approach. Given this, I suggest: i) explaining how this limitation affects interpretation of the findings, ii) providing more detail about the characteristics of the two participating organizations, and iii) reflecting on potential selection biases, particularly considering that the study was commissioned by the organizations themselves. | i) We have reworded the limitation to state that the absence of demographic data for most participants prevents “the opportunity to conduct intersectional analysis” (as opposed to “limiting opportunities for interactional analysis”).  ii) We have added a new “Study Setting” subsection (p. 10 lines 217-225) which provides further information on the two participating organizations, describing them as large regional health organizations within a publicly funded provincial health system that deliver integrated mental health and substance use services across urban centres and surrounding rural communities. This added context is intended to support assessment of transferability while maintaining organizational anonymity.  iiI) We have already addressed this concern in the Limitations section where we note: “As part of our recruitment process, and to ensure transparency, we disclosed that this study was commissioned by the health organizations, and that results would be communicated directly to it. Consequently, we may have not reached those experiencing the greatest structural vulnerabilities when interacting with health systems, or those whose experiences of trauma during their interactions with health systems would have discouraged them from participating in a study involving those health organizations”. |
| Rigor of the thematic analysis The description of the thematic analysis is too general to adequately assess interpretive rigor. It would be valuable to: i) include examples of codes, subthemes, and analytic decisions, ii) explain how data from participants with lived experience and providers were integrated or contrasted, and iii) incorporate an explicit reflexivity section, given the sensitivity of the topic and the composition of the research team.  For instance, the manuscript notes: “Two members systematically and independently reviewed transcripts…”, but does not detail how discrepancies were resolved or how interpretive coherence was ensured. | i) We have now included this on pp. 16-18 lines 341-347  ii) We now state that “ To centre the voice of PWLLE, the focus group transcripts were coded first, followed by coding the interview transcripts.” (p. 15 lines 314-315) and we note “Final codes reflected repeated elements across the dataset while preserving the nuances of diverse perspectives. For example, PWLLE and clinicians described involuntary treatment as stressful or uncomfortable, but their interpretations diverged: clinicians emphasized safety, legality, and resource constraints, whereas PWLLE highlighted loss of autonomy and emotional distress.” (p. 15 lines 319-323)  iii) We already include a Reflexivity section (pp. 9-10 lines 182-215) in which we note that “Analytic rigour was supported through iterative coding, regular analytic meetings, and collaborative discussion of emerging interpretations. Preliminary themes were reviewed with the Advisory Committee, and areas of divergence or uncertainty were discussed with attention to how positionality, professional roles, and lived experience shaped meaning-making.”  We have also revised terminology throughout this section to improve conceptual precision. Specifically, we replaced “coding discrepancies” to “interpretative differences” to better reflect that variations in coding were treated as differences in analytical perspective rather than deviations from a single correct interpretation. We also replaced “reach consensus” with “develop a shared analytic understanding” to more accurately reflect our inductive and interpretive approach, which was not aimed at establishing intercoder reliability but at collaboratively refining codes to reflect participants’ accounts. We believe these revisions strengthen clarity regarding the interpretive and reflexive nature of the analysis. |
| Scope of the conclusions Some statements extend beyond what the data can support. For example:  “Overall, involuntary psychiatric admission and treatment under the MH Act is not experienced as consistent with the principles of equity-oriented care.”  I recommend tempering such conclusions, anchoring them explicitly in the study’s data, and acknowledging the limitations of qualitative designs for generalization. | The phrase cited by the reviewer (“Overall, involuntary psychiatric admission and treatment under the MH Act is not experienced as consistent with the principles of equity-oriented care”) was from an earlier version of the manuscript. In our revised submission, we updated this sentence to state: “In summary, our findings demonstrate that, overall, participants in this study did not experience involuntary psychiatric admission and treatment under the MH Act in ways that are consistent with the principles of equity-oriented care.” (p. 40 lines 813-817) We chose this revised wording specifically to clarify that this interpretation is grounded in the experiences of study participants and is not intended to imply generalizability beyond the study sample. |
| Incremental contribution. The manuscript engages appropriately with existing literature; however, it would be helpful to articulate more clearly: i) what this study contributes that has not been previously documented, ii) how provider perspectives complement or challenge existing evidence, and iii) the extent to which the participatory approach yields novel insights. | We have added a paragraph to the Discussion (p. 41 lines 838-846) to more clearly articulate the study’s incremental contribution:   - while many of the substantive findings are consistent with existing literature, this study contributes context-specific evidence from a “deemed consent” legal framework within two large regional health organizations operating across urban and rural settings. - inclusion of multiple perspectives enables a more integrated understanding of involuntary mental health care across roles within the system, highlighting both shared and divergent experiences. - participatory design strengthened the contextual grounding and interpretive depth of the analysis through involvement of people with lived experience in data collection and interpretation. |
| Review minor typographical errors (e.g., “an 2021”). | We have corrected a few minor typographical errors. |
| Clarify whether interview and focus group guides can be included as supplementary material. | Interview and focus group guides are now available as supplementary material. |
| Consider balancing the number of quotations from participants with lived experience and providers across themes. | We have carefully reviewed the distribution of quotations across participant groups and made targeted revisions to improve balance and representation across themes. Specifically, we have added an additional quotation from a PWLLE participant within Theme 6 to strengthen the inclusion of lived experience perspectives in this section (p. 35 lines 712-715). We have also clarified role-differentiated contributions in Themes 5 and 7 specifying when issues were primarily raised by clinicians and service providers, reflecting the structure of the dataset rather than selective emphasis. Together, these revisions improve transparency regarding the distribution of perspectives while preserving the analytic integrity of each theme. |
