# Supplementary material for: “Backed into a Corner”: Lived experiences of receiving and providing involuntary psychiatric treatment under British Columbia’s Mental Health Act
Source: PLoS One. 2026 Jun 10;21(6):e0329049. doi: 10.1371/journal.pone.0329049 (PMC13252785; doi:10.1371/journal.pone.0329049)
Supplement: Peer review history

# Peer review: Decision Letter 0

**DOI:** 10.1371/journal.pone.0329049.r001

**Authors:** Felipe Agudelo-Hernández

5 Nov 2025

PONE-D-25-37120

"Backed into a Corner": Lived experiences of receiving and providing involuntary psychiatric treatment under British Columbia's Mental Health Act

PLOS ONE

Dear Dr. Banjo,

Thank you for submitting your manuscript to PLOS ONE. After careful consideration, we feel that it has merit but does not fully meet PLOS ONE’s publication criteria as it currently stands. Therefore, we invite you to submit a revised version of the manuscript that addresses the points raised during the review process.

Please submit your revised manuscript by Dec 20 2025 11:59PM. If you will need more time than this to complete your revisions, please reply to this message or contact the journal office at plosone@plos.org. When you're ready to submit your revision, log on to https://www.editorialmanager.com/pone/ and select the 'Submissions Needing Revision' folder to locate your manuscript file.

Please include the following items when submitting your revised manuscript:

- A rebuttal letter that responds to each point raised by the academic editor and reviewer(s). You should upload this letter as a separate file labeled 'Response to Reviewers'.
- A marked-up copy of your manuscript that highlights changes made to the original version. You should upload this as a separate file labeled 'Revised Manuscript with Track Changes'.
- An unmarked version of your revised paper without tracked changes. You should upload this as a separate file labeled 'Manuscript'.

If you would like to make changes to your financial disclosure, please include your updated statement in your cover letter. Guidelines for resubmitting your figure files are available below the reviewer comments at the end of this letter.

If applicable, we recommend that you deposit your laboratory protocols in protocols.io to enhance the reproducibility of your results. Protocols.io assigns your protocol its own identifier (DOI) so that it can be cited independently in the future. For instructions see: https://journals.plos.org/plosone/s/submission-guidelines#loc-laboratory-protocols. Additionally, PLOS ONE offers an option for publishing peer-reviewed Lab Protocol articles, which describe protocols hosted on protocols.io. Read more information on sharing protocols at https://plos.org/protocols?utm_medium=editorial-email&utm_source=authorletters&utm_campaign=protocols.

We look forward to receiving your revised manuscript.

Kind regards,

Felipe Agudelo-Hernández, Ph.D, M.D

Academic Editor

PLOS ONE

Journal Requirements:

When submitting your revision, we need you to address these additional requirements.

1. Please ensure that your manuscript meets PLOS ONE's style requirements, including those for file naming. The PLOS ONE style templates can be found at

https://journals.plos.org/plosone/s/file?id=wjVg/PLOSOne_formatting_sample_main_body.pdf and

https://journals.plos.org/plosone/s/file?id=ba62/PLOSOne_formatting_sample_title_authors_affiliations.pdf

2. In this instance it seems there may be acceptable restrictions in place that prevent the public sharing of your minimal data. However, in line with our goal of ensuring long-term data availability to all interested researchers, PLOS’ Data Policy states that authors cannot be the sole named individuals responsible for ensuring data access (http://journals.plos.org/plosone/s/data-availability#loc-acceptable-data-sharing-methods).

Data requests to a non-author institutional point of contact, such as a data access or ethics committee, helps guarantee long term stability and availability of data. Providing interested researchers with a durable point of contact ensures data will be accessible even if an author changes email addresses, institutions, or becomes unavailable to answer requests.

Before we proceed with your manuscript, please also provide non-author contact information (phone/email/hyperlink) for a data access committee, ethics committee, or other institutional body to which data requests may be sent. If no institutional body is available to respond to requests for your minimal data, please consider if there any institutional representatives who did not collaborate in the study, and are not listed as authors on the manuscript, who would be able to hold the data and respond to external requests for data access? If so, please provide their contact information (i.e., email address). Please also provide details on how you will ensure persistent or long-term data storage and availability.

3. If the reviewer comments include a recommendation to cite specific previously published works, please review and evaluate these publications to determine whether they are relevant and should be cited. There is no requirement to cite these works unless the editor has indicated otherwise.

Additional Editor Comments:

Dear authors

Thank you for allowing us to review your manuscript. Below, I offer some suggestions that could clarify and strengthen the manuscript, in addition to the points raised by the reviewers:

- The information on the implementation of the Convention on the Rights of Persons with Disabilities (CRPD) could be expanded. The following articles/authors are recommended:

Davidson L. (2025). Swinging the pendulum from 'a necessary evil' to 'the dignity of risk': Can new UN legislative guidance help to end psychiatric coercion? International Journal of Law and Psychiatry, 102, 102102. https://doi.org/10.1016/j.ijlp.2025.102102.

Szmukler G. (2019). "Capacity", "best interests", "will and preferences" and the UN Convention on the Rights of Persons with Disabilities. World Psychiatry: Official Journal of the World Psychiatric Association (WPA), 18(1), 34–41. https://doi.org/10.1002/wps.20584

- Other types of coercive practices or involuntary treatment that are less evident in care processes could also be mentioned, such as ignoring people's wishes, not including them in treatment plans, etc.

In the methods and results section, it is advisable to adhere to the COREQ criteria (https://resources.equator-network.org/reporting-guidelines/coreq/).

R1:

This manuscript offers a valuable and timely contribution to the international debate on coercive practices in mental health, by rigorously and sensitively documenting the lived experiences of involuntary treatment. I recommend strengthening the discussion on voluntary alternatives and community-based care models, as well as expanding the intersectional analysis to further enrich the equity perspective that guides the study. It is also necessary to more explicitly acknowledge the limitations of generalizability in the discussion, as well as to include a more robust section on possible solutions or successful alternative models.

R2:

Major Comments

1. Conceptual framing and theoretical depth

While the manuscript refers to “equity-oriented care,” “trauma- and violence-informed,” “culturally safe,” and “anti-racist” approaches, these concepts are not clearly defined nor theoretically integrated. The paper risks presenting them as buzzwords rather than analytic lenses.

Recommendation: Clarify how these frameworks informed the design, data collection, and interpretation. Explain whether the analysis was guided by a specific theoretical approach (e.g., intersectionality, critical mental health studies, decolonial theory). A brief conceptual framework section early in the paper would strengthen the argument substantially.

2. Methodological rigor and reflexivity

Although the participatory design is commendable, the description of how reflexivity, positionality, and analytic rigor were maintained is insufficient.

• How were power dynamics between researchers, PWLLE, and institutional staff navigated?

• Was there member checking or peer debriefing?

• How were divergent interpretations handled?

Recommendation: Include a Reflexivity and Rigor subsection explaining how the research team’s composition and lived experiences shaped the analysis. Consider adding a short table summarizing strategies for ensuring credibility and trustworthiness (e.g., triangulation, iterative coding, Advisory Committee validation).

3. Data analysis and interpretation

Thematic analysis is appropriate, but the resulting themes read as extended summaries rather than interpretive insights. The manuscript currently catalogs participants’ experiences but stops short of offering a conceptual synthesis or theoretical contribution.

Example: The “all-or-nothing consent” theme could be deepened using frameworks of biopower, governance, or medical paternalism.

Similarly, the “lack of voluntary options” theme could be connected to neoliberal health reforms or deinstitutionalization failures.

Recommendation: Move beyond description. Clarify what these findings mean for understanding coercion and equity, not just that they exist. Consider developing a conceptual figure linking structural determinants, coercive practices, and experiential outcomes.

4. Ethical and epistemic considerations

The study addresses deeply traumatic experiences. While it notes ethical approval, it lacks reflection on how risks of re-traumatization, coercion, or confidentiality were mitigated.

Recommendation: Add an Ethical Considerations section discussing the emotional risks for participants, how informed consent was practiced meaningfully in a coercive context, and how peer facilitators were supported.

5. Discussion and originality

The discussion largely repeats results and lists literature that confirms them, without building a distinctive argument.

Recommendation: Reorganize the discussion into three clear layers:

• Empirical: What this study adds to the evidence base in BC.

• Theoretical: How it extends debates on coercion, rights, and structural determinants.

• Policy/Practice: Concrete implications for reform (e.g., alternatives to involuntary detention, peer inclusion models, community-based prevention).

The paper could also more explicitly connect its findings to Canada’s obligations under the UN Convention on the Rights of Persons with Disabilities, analyzing not only that contradictions exist but how and why they persist.

Minor Comments

Participant demographics are extremely limited. Even minimal data (gender, ethnicity, years since admission) would improve transparency.

Several sections could be condensed by removing duplicated findings in Results and Discussion

[Note: HTML markup is below. Please do not edit.]

Reviewers' comments:

Reviewer's Responses to Questions

**Comments to the Author**

1. Is the manuscript technically sound, and do the data support the conclusions?

The manuscript must describe a technically sound piece of scientific research with data that supports the conclusions. Experiments must have been conducted rigorously, with appropriate controls, replication, and sample sizes. The conclusions must be drawn appropriately based on the data presented.

Reviewer #1: Yes

Reviewer #2: Yes

**********

2. Has the statistical analysis been performed appropriately and rigorously?

Reviewer #1: Yes

Reviewer #2: Yes

**********

3. Have the authors made all data underlying the findings in their manuscript fully available?

The PLOS Data policy requires authors to make all data underlying the findings described in their manuscript fully available without restriction, with rare exception (please refer to the Data Availability Statement in the manuscript PDF file). The data should be provided as part of the manuscript or its supporting information, or deposited to a public repository. For example, in addition to summary statistics, the data points behind means, medians and variance measures should be available. If there are restrictions on publicly sharing data—e.g. participant privacy or use of data from a third party—those must be specified.

Reviewer #1: Yes

Reviewer #2: Yes

**********

4. Is the manuscript presented in an intelligible fashion and written in standard English?

PLOS ONE does not copyedit accepted manuscripts, so the language in submitted articles must be clear, correct, and unambiguous. Any typographical or grammatical errors should be corrected at revision, so please note any specific errors here.

Reviewer #1: Yes

Reviewer #2: Yes

**********

5. Review Comments to the Author

Please use the space provided to explain your answers to the questions above. You may also include additional comments for the author, including concerns about dual publication, research ethics, or publication ethics. (Please upload your review as an attachment if it exceeds 20,000 characters)

Reviewer #1: This manuscript offers a valuable and timely contribution to the international debate on coercive practices in mental health, by rigorously and sensitively documenting the lived experiences of involuntary treatment. I recommend strengthening the discussion on voluntary alternatives and community-based care models, as well as expanding the intersectional analysis to further enrich the equity perspective that guides the study. It is also necessary to more explicitly acknowledge the limitations of generalizability in the discussion, as well as to include a more robust section on possible solutions or successful alternative models.

Reviewer #2: Major Comments

1. Conceptual framing and theoretical depth

While the manuscript refers to “equity-oriented care,” “trauma- and violence-informed,” “culturally safe,” and “anti-racist” approaches, these concepts are not clearly defined nor theoretically integrated. The paper risks presenting them as buzzwords rather than analytic lenses.

Recommendation: Clarify how these frameworks informed the design, data collection, and interpretation. Explain whether the analysis was guided by a specific theoretical approach (e.g., intersectionality, critical mental health studies, decolonial theory). A brief conceptual framework section early in the paper would strengthen the argument substantially.

2. Methodological rigor and reflexivity

Although the participatory design is commendable, the description of how reflexivity, positionality, and analytic rigor were maintained is insufficient.

• How were power dynamics between researchers, PWLLE, and institutional staff navigated?

• Was there member checking or peer debriefing?

• How were divergent interpretations handled?

Recommendation: Include a Reflexivity and Rigor subsection explaining how the research team’s composition and lived experiences shaped the analysis. Consider adding a short table summarizing strategies for ensuring credibility and trustworthiness (e.g., triangulation, iterative coding, Advisory Committee validation).

3. Data analysis and interpretation

Thematic analysis is appropriate, but the resulting themes read as extended summaries rather than interpretive insights. The manuscript currently catalogs participants’ experiences but stops short of offering a conceptual synthesis or theoretical contribution.

Example: The “all-or-nothing consent” theme could be deepened using frameworks of biopower, governance, or medical paternalism.

Similarly, the “lack of voluntary options” theme could be connected to neoliberal health reforms or deinstitutionalization failures.

Recommendation: Move beyond description. Clarify what these findings mean for understanding coercion and equity, not just that they exist. Consider developing a conceptual figure linking structural determinants, coercive practices, and experiential outcomes.

4. Ethical and epistemic considerations

The study addresses deeply traumatic experiences. While it notes ethical approval, it lacks reflection on how risks of re-traumatization, coercion, or confidentiality were mitigated.

Recommendation: Add an Ethical Considerations section discussing the emotional risks for participants, how informed consent was practiced meaningfully in a coercive context, and how peer facilitators were supported.

5. Discussion and originality

The discussion largely repeats results and lists literature that confirms them, without building a distinctive argument.

Recommendation: Reorganize the discussion into three clear layers:

• Empirical: What this study adds to the evidence base in BC.

• Theoretical: How it extends debates on coercion, rights, and structural determinants.

• Policy/Practice: Concrete implications for reform (e.g., alternatives to involuntary detention, peer inclusion models, community-based prevention).

The paper could also more explicitly connect its findings to Canada’s obligations under the UN Convention on the Rights of Persons with Disabilities, analyzing not only that contradictions exist but how and why they persist.

Minor Comments

Participant demographics are extremely limited. Even minimal data (gender, ethnicity, years since admission) would improve transparency.

Several sections could be condensed by removing duplicated findings in Results and Discussion.

**********

6. PLOS authors have the option to publish the peer review history of their article (what does this mean?). If published, this will include your full peer review and any attached files.

If you choose “no”, your identity will remain anonymous but your review may still be made public.

**Do you want your identity to be public for this peer review?** For information about this choice, including consent withdrawal, please see our Privacy Policy.

Reviewer #1: **Yes:** Laura Ines Plata-Casas

Reviewer #2: No

**********

[NOTE: If reviewer comments were submitted as an attachment file, they will be attached to this email and accessible via the submission site. Please log into your account, locate the manuscript record, and check for the action link "View Attachments". If this link does not appear, there are no attachment files.]

To ensure your figures meet our technical requirements, please review our figure guidelines: https://journals.plos.org/plosone/s/figures

You may also use PLOS’s free figure tool, NAAS, to help you prepare publication quality figures: https://journals.plos.org/plosone/s/figures#loc-tools-for-figure-preparation.

NAAS will assess whether your figures meet our technical requirements by comparing each figure against our figure specifications.

Attachment Submitted filename: Reviewer Report.docx

# Author comment: Author response to Decision Letter 1

**DOI:** 10.1371/journal.pone.0329049.r002

20 Dec 2025

We have provided a detailed, point-by-point response to all reviewer and editor comments in the attached “Response to Reviewers” document. In brief, we revised the manuscript to strengthen conceptual framing, reflexivity and methodological transparency, analytic depth in the Results, and synthesis in the Discussion, and to address ethical and data availability requirements. Page and line numbers are provided in the attached response to indicate where changes were made.

Attachment Submitted filename: 2025-12-20 Response to Reviewers.docx

# Peer review: Decision Letter 1

**DOI:** 10.1371/journal.pone.0329049.r003

**Authors:** Colleen Dell

24 Feb 2026

PONE-D-25-37120R1

"Backed into a Corner": Lived experiences of receiving and providing involuntary psychiatric treatment under British Columbia's Mental Health Act

PLOS One

Dear Dr. Banjo,

Thank you for submitting your manuscript to PLOS ONE. After careful consideration, we feel that it has merit but does not fully meet PLOS ONE’s publication criteria as it currently stands. Therefore, we invite you to submit a revised version of the manuscript that addresses the points raised during the review process.

==============================

**Please see additional reviewer suggestions attached.**

==============================

Please submit your revised manuscript by Apr 10 2026 11:59PM. If you will need more time than this to complete your revisions, please reply to this message or contact the journal office at plosone@plos.org. There is no problem with having more time if it is required. When you're ready to submit your revision, log on to https://www.editorialmanager.com/pone/ and select the 'Submissions Needing Revision' folder to locate your manuscript file.

Please include the following items when submitting your revised manuscript:

- A letter that responds to each point raised by the academic editor and reviewer(s). You should upload this letter as a separate file labeled 'Response to Reviewers'.
- A marked-up copy of your manuscript that highlights changes made to the original version. You should upload this as a separate file labeled 'Revised Manuscript with Track Changes'.
- An unmarked version of your revised paper without tracked changes. You should upload this as a separate file labeled 'Manuscript'.

If you would like to make changes to your financial disclosure, please include your updated statement in your cover letter. Guidelines for resubmitting your figure files are available below the reviewer comments at the end of this letter.

If applicable, we recommend that you deposit your laboratory protocols in protocols.io to enhance the reproducibility of your results. Protocols.io assigns your protocol its own identifier (DOI) so that it can be cited independently in the future. For instructions see: https://journals.plos.org/plosone/s/submission-guidelines#loc-laboratory-protocols. Additionally, PLOS ONE offers an option for publishing peer-reviewed Lab Protocol articles, which describe protocols hosted on protocols.io. Read more information on sharing protocols at https://plos.org/protocols?utm_medium=editorial-email&utm_source=authorletters&utm_campaign=protocols.

We look forward to receiving your revised manuscript.

Kind regards,

Colleen Anne Dell, Ph.D.

Academic Editor

PLOS One

Journal Requirements:

If the reviewer comments include a recommendation to cite specific previously published works, please review and evaluate these publications to determine whether they are relevant and should be cited. There is no requirement to cite these works unless the editor has indicated otherwise.

Additional Editor Comments:

Dear Authors of the manuscript titled "Backed into a Corner": Lived experiences of receiving and providing involuntary psychiatric treatment under British Columbia's Mental Health Act",

I am coming on as a new Editor in the manuscript review process, for which initial reviewer #1 is asking for major revisions (details attached) and reviewer #2 is also suggesting methodological rigour could be more transparent. Please attend to the required revision for further consideration to be published in PLOS One.

Sincerely, Colleen

[Note: HTML markup is below. Please do not edit.]

Reviewers' comments:

Reviewer's Responses to Questions

**Comments to the Author**

1. If the authors have adequately addressed your comments raised in a previous round of review and you feel that this manuscript is now acceptable for publication, you may indicate that here to bypass the “Comments to the Author” section, enter your conflict of interest statement in the “Confidential to Editor” section, and submit your "Accept" recommendation.

Reviewer #1: All comments have been addressed

Reviewer #2: All comments have been addressed

**********

2. Is the manuscript technically sound, and do the data support the conclusions?

The manuscript must describe a technically sound piece of scientific research with data that supports the conclusions. Experiments must have been conducted rigorously, with appropriate controls, replication, and sample sizes. The conclusions must be drawn appropriately based on the data presented.

Reviewer #1: Yes

Reviewer #2: Yes

**********

3. Has the statistical analysis been performed appropriately and rigorously?

Reviewer #1: Yes

Reviewer #2: Yes

**********

4. Have the authors made all data underlying the findings in their manuscript fully available?

The PLOS Data policy requires authors to make all data underlying the findings described in their manuscript fully available without restriction, with rare exception (please refer to the Data Availability Statement in the manuscript PDF file). The data should be provided as part of the manuscript or its supporting information, or deposited to a public repository. For example, in addition to summary statistics, the data points behind means, medians and variance measures should be available. If there are restrictions on publicly sharing data—e.g. participant privacy or use of data from a third party—those must be specified.

Reviewer #1: (No Response)

Reviewer #2: Yes

**********

5. Is the manuscript presented in an intelligible fashion and written in standard English?

PLOS ONE does not copyedit accepted manuscripts, so the language in submitted articles must be clear, correct, and unambiguous. Any typographical or grammatical errors should be corrected at revision, so please note any specific errors here.

Reviewer #1: Yes

Reviewer #2: Yes

**********

6. Review Comments to the Author

Please use the space provided to explain your answers to the questions above. You may also include additional comments for the author, including concerns about dual publication, research ethics, or publication ethics. (Please upload your review as an attachment if it exceeds 20,000 characters)

Reviewer #1: I appreciate the opportunity to review this manuscript, which addresses a critical topic of high ethical sensitivity. The study is relevant and presents important findings regarding the experiences of individuals who receive and provide involuntary treatment under British Columbia’s Mental Health Act. Below, I offer observations intended to strengthen the clarity, methodological rigor, and overall contribution of the work.

1. Clarity of the objectives and conceptual framework The study’s objectives are broad and combine description, critical analysis, and policy-oriented recommendations. It would be helpful to clarify: i) the specific research question(s), ii) how the “equity-oriented care” framework was operationalized within the analysis, and iii) the distinction between empirical findings and normative positions.

For example, the manuscript states: “Our aims were: (1) to document and examine, from an equity-oriented perspective…”, yet it does not describe how this framework guided coding, interpretation, or theme development.

2. Sampling, sample composition, and transferability The absence of demographic data for most participants with lived experience limits the possibility of conducting an intersectional analysis, despite the manuscript’s emphasis on this approach. Given this, I suggest: i) explaining how this limitation affects interpretation of the findings, ii) providing more detail about the characteristics of the two participating organizations, and iii) reflecting on potential selection biases, particularly considering that the study was commissioned by the organizations themselves.

3. Rigor of the thematic analysis The description of the thematic analysis is too general to adequately assess interpretive rigor. It would be valuable to: i) include examples of codes, subthemes, and analytic decisions, ii) explain how data from participants with lived experience and providers were integrated or contrasted, and iii) incorporate an explicit reflexivity section, given the sensitivity of the topic and the composition of the research team.

For instance, the manuscript notes: “Two members systematically and independently reviewed transcripts…”, but does not detail how discrepancies were resolved or how interpretive coherence was ensured.

4. Scope of the conclusions Some statements extend beyond what the data can support. For example:

“Overall, involuntary psychiatric admission and treatment under the MH Act is not experienced as consistent with the principles of equity-oriented care.”

I recommend tempering such conclusions, anchoring them explicitly in the study’s data, and acknowledging the limitations of qualitative designs for generalization.

5. Incremental contribution The manuscript engages appropriately with existing literature; however, it would be helpful to articulate more clearly: i) what this study contributes that has not been previously documented, ii) how provider perspectives complement or challenge existing evidence, and iii) the extent to which the participatory approach yields novel insights.

Minor comments

• Review minor typographical errors (e.g., “an 2021”).

• Clarify whether interview and focus group guides can be included as supplementary material.

• Consider balancing the number of quotations from participants with lived experience and providers across themes.

• The title is evocative, but it may be useful to clarify that the phrase originates from providers rather than patients.

The manuscript has significant potential and addresses an issue of substantial social and ethical importance. With a major revision that strengthens methodological transparency, reflexivity, and interpretive precision, it could become a valuable contribution to the literature on psychiatric coercion and equity in mental health.

Reviewer #2: Dear Dr. Banjo and colleagues,

Thank you for the opportunity to review the revised version of your manuscript. I appreciate the thoughtful and substantive way in which you engaged with the previous feedback. The revisions have clearly strengthened the paper.

The addition of a clear conceptual framework significantly improves the manuscript. Equity-oriented care is now presented as an analytic lens rather than a set of descriptors, and the integration of structural determinants, colonialism, and power dynamics provides stronger theoretical grounding. The study’s contribution to debates on coercion and structural inequities is much clearer.

Methodologically, the new Reflexivity and Rigour and Ethical Considerations sections enhance transparency and credibility. Your discussion of participatory processes, power dynamics, and trauma-informed safeguards meaningfully addresses prior concerns. The justification for limited demographic data is also well explained.

The analysis is now more interpretive, particularly in linking participants’ experiences to systemic constraints and institutional logics. While some themes remain slightly more descriptive than others, the overall analytic depth has improved considerably. A final tightening of thematic synthesis paragraphs could further enhance coherence.

The Discussion is more cohesive and layered, with clearer policy and structural implications. A brief explicit statement of the manuscript’s theoretical contribution, and, if feasible, a short link to Canada’s human rights obligations, could further sharpen the paper’s impact.

Overall, this is a substantially improved manuscript that makes an important and timely contribution to scholarship on involuntary psychiatric treatment and equity-oriented mental health reform.

Thank you again for the opportunity to review this meaningful work.

**********

7. PLOS authors have the option to publish the peer review history of their article (what does this mean?). If published, this will include your full peer review and any attached files.

If you choose “no”, your identity will remain anonymous but your review may still be made public.

**Do you want your identity to be public for this peer review?** For information about this choice, including consent withdrawal, please see our Privacy Policy.

Reviewer #1: **Yes:** Laura Ines Plata-Casas

Reviewer #2: No

**********

[NOTE: If reviewer comments were submitted as an attachment file, they will be attached to this email and accessible via the submission site. Please log into your account, locate the manuscript record, and check for the action link "View Attachments". If this link does not appear, there are no attachment files.]

To ensure your figures meet our technical requirements, please review our figure guidelines: https://journals.plos.org/plosone/s/figures

You may also use PLOS’s free figure tool, NAAS, to help you prepare publication quality figures: https://journals.plos.org/plosone/s/figures#loc-tools-for-figure-preparation.

NAAS will assess whether your figures meet our technical requirements by comparing each figure against our figure specifications.

Attachment Submitted filename: Reviewer Report.pdf

# Author comment: Author response to Decision Letter 2

**DOI:** 10.1371/journal.pone.0329049.r004

22 Apr 2026

We have provided a detailed point-by-point response to all reviewer and editor comments in the attached document. Corresponding revisions have been incorporated into the revised manuscript.

Attachment Submitted filename: 2026-04-22 Response to Reviewers.docx

# Peer review: Decision Letter 2

**DOI:** 10.1371/journal.pone.0329049.r005

**Authors:** Andrea Knittel

26 May 2026

"Backed into a Corner": Lived experiences of receiving and providing involuntary psychiatric treatment under British Columbia's Mental Health Act

PONE-D-25-37120R2

Dear Dr. Banjo,

We’re pleased to inform you that your manuscript has been judged scientifically suitable for publication and will be formally accepted for publication once it meets all outstanding technical requirements.

Within one week, you’ll receive an e-mail detailing the required amendments. When these have been addressed, you’ll receive a formal acceptance letter and your manuscript will be scheduled for publication.

An invoice will be generated when your article is formally accepted. Please note, if your institution has a publishing partnership with PLOS and your article meets the relevant criteria, all or part of your publication costs will be covered. Please make sure your user information is up-to-date by logging into Editorial Manager at Editorial Manager® and clicking the ‘Update My Information' link at the top of the page. For questions related to billing, please contact billing support.

If your institution or institutions have a press office, please notify them about your upcoming paper to help maximize its impact. If they’ll be preparing press materials, please inform our press team as soon as possible -- no later than 48 hours after receiving the formal acceptance. Your manuscript will remain under strict press embargo until 2 pm Eastern Time on the date of publication. For more information, please contact onepress@plos.org.

Kind regards,

Andrea K. Knittel

Academic Editor

PLOS One

Additional Editor Comments (optional):

Reviewers' comments:

Reviewer's Responses to Questions

**Comments to the Author**

1. If the authors have adequately addressed your comments raised in a previous round of review and you feel that this manuscript is now acceptable for publication, you may indicate that here to bypass the “Comments to the Author” section, enter your conflict of interest statement in the “Confidential to Editor” section, and submit your "Accept" recommendation.

Reviewer #1: All comments have been addressed

Reviewer #2: All comments have been addressed

**********

2. Is the manuscript technically sound, and do the data support the conclusions?

The manuscript must describe a technically sound piece of scientific research with data that supports the conclusions. Experiments must have been conducted rigorously, with appropriate controls, replication, and sample sizes. The conclusions must be drawn appropriately based on the data presented.

Reviewer #1: Yes

Reviewer #2: Yes

**********

3. Has the statistical analysis been performed appropriately and rigorously?

Reviewer #1: Yes

Reviewer #2: Yes

**********

4. Have the authors made all data underlying the findings in their manuscript fully available?

The PLOS Data policy requires authors to make all data underlying the findings described in their manuscript fully available without restriction, with rare exception (please refer to the Data Availability Statement in the manuscript PDF file). The data should be provided as part of the manuscript or its supporting information, or deposited to a public repository. For example, in addition to summary statistics, the data points behind means, medians and variance measures should be available. If there are restrictions on publicly sharing data—e.g. participant privacy or use of data from a third party—those must be specified.

Reviewer #1: Yes

Reviewer #2: Yes

**********

5. Is the manuscript presented in an intelligible fashion and written in standard English?

PLOS ONE does not copyedit accepted manuscripts, so the language in submitted articles must be clear, correct, and unambiguous. Any typographical or grammatical errors should be corrected at revision, so please note any specific errors here.

Reviewer #1: Yes

Reviewer #2: Yes

**********

6. Review Comments to the Author

Please use the space provided to explain your answers to the questions above. You may also include additional comments for the author, including concerns about dual publication, research ethics, or publication ethics. (Please upload your review as an attachment if it exceeds 20,000 characters)

Reviewer #1: The revised manuscript reflects meaningful engagement with the feedback provided during the previous review cycle

Reviewer #2: I believe this article should now be accepted and published. The manuscript is presented in an intelligible fashion and all the data analysis appears to be accurate.

**********

7. PLOS authors have the option to publish the peer review history of their article (what does this mean?). If published, this will include your full peer review and any attached files.

If you choose “no”, your identity will remain anonymous but your review may still be made public.

**Do you want your identity to be public for this peer review?** For information about this choice, including consent withdrawal, please see our Privacy Policy.

Reviewer #1: No

Reviewer #2: **Yes:** Lina Cuadrado

**********

# Editor report: Acceptance letter

**DOI:** 10.1371/journal.pone.0329049.r006

**Authors:** Andrea Knittel

PONE-D-25-37120R2

PLOS One

Dear Dr. Banjo,

I'm pleased to inform you that your manuscript has been deemed suitable for publication in PLOS One. Congratulations! Your manuscript is now being handed over to our production team.

At this stage, our production department will prepare your paper for publication. This includes ensuring the following:

* All references, tables, and figures are properly cited

* All relevant supporting information is included in the manuscript submission,

* There are no issues that prevent the paper from being properly typeset

You will receive further instructions from the production team, including instructions on how to review your proof when it is ready. Please keep in mind that we are working through a large volume of accepted articles, so please give us a few days to review your paper and let you know the next and final steps.

Lastly, if your institution or institutions have a press office, please let them know about your upcoming paper now to help maximize its impact. If they'll be preparing press materials, please inform our press team within the next 48 hours. Your manuscript will remain under strict press embargo until 2 pm Eastern Time on the date of publication. For more information, please contact onepress@plos.org.

You will receive an invoice from PLOS for your publication fee after your manuscript has reached the completed accept phase. If you receive an email requesting payment before acceptance or for any other service, this may be a phishing scheme. Learn how to identify phishing emails and protect your accounts at https://explore.plos.org/phishing.

If we can help with anything else, please email us at customercare@plos.org.

Thank you for submitting your work to PLOS ONE and supporting open access.

Kind regards,

PLOS ONE Editorial Office Staff

on behalf of

Dr. Andrea K. Knittel

Academic Editor

PLOS One
